# An anti-EpCAM antibody EpAb2-6 for the treatment of colon cancer

**DOI:** 10.18632/oncotarget.4453

**Published:** 2015-08-06

**Authors:** Mei-Ying Liao, Jun-Kai Lai, Mark Yen-Ping Kuo, Ruei-Min Lu, Cheng-Wei Lin, Ping-Chang Cheng, Kang-Hao Liang, Han-Chung Wu

**Affiliations:** ^1^ Institute of Cellular and Organismic Biology, Academia Sinica, Taipei, Taiwan; ^2^ Graduate Institute of Clinical Dentistry, School of Dentistry, National Taiwan University, Taipei, Taiwan; ^3^ Genomics Research Center, Academia Sinica, Taipei, Taiwan

**Keywords:** colorectal carcinoma, EpCAM, therapeutic antibody, targeting imaging, cancer therapy

## Abstract

Epithelial cell adhesion molecule (EpCAM) is known to be overexpressed in epithelial cancers associated with enhanced malignant potential, particularly colorectal carcinoma (CRC) and head and neck squamous cell carcinoma (HNSCC). However, it is unknown whether progression of malignance can be directly inhibited by targeting EpCAM. Here, we have generated five novel monoclonal antibodies (mAbs) against EpCAM. One of these anti-EpCAM mAbs, EpAb2-6, was found to induce cancer cell apoptosis *in vitro*, inhibit tumor growth, and prolong the overall survival of both a pancreatic cancer metastatic mouse model and mice with human colon carcinoma xenografts. EpAb2-6 also increases the therapeutic efficacy of irinotecan, fluorouracil, and leucovorin (IFL) therapy in a colon cancer animal model and gemcitabine therapy in a pancreatic cancer animal model. Furthermore, EpAb2-6, which binds to positions Y95 and D96 of the EGF-II/TY domain of EpCAM, inhibits production of EpICD, thereby decreasing its translocation and subsequent signal activation. Collectively, our results indicate that the novel anti-EpCAM mAb can potentially be used for cancer-targeted therapy.

## INTRODUCTION

Epithelial cell adhesion molecule (EpCAM; CD326) is a 39 kDa type I transmembrane glycoprotein, encoded by the *TACSTD1* gene (located on the long arm of chromosome 2p21). EpCAM is known to be overexpressed in epithelial cancers associated with enhanced proliferation, invasion, metastasis, malignant potential, chemo-/radioresistance, and decreased overall survival of cancer patients [1–4]. Recent data suggest a more multipotent role of EpCAM in cell-cell adhesion, cell signaling, migration, and differentiation [5]. As it is frequently highly expressed in tumor tissues and metastatic cancer cells in transit via blood or lymphatic vessels [3, 6, 7], EpCAM has gained attention as a potential target for diagnostic and antibody-based immunotherapies for a spectrum of malignancies [6, 8–11].

The first mAb ever used in human cancer therapy was a murine IgG2a antibody (Edrecolomab; Panorex; mAb 17-1A) directed against EpCAM [12]. Edrecolomab was approved in Germany in 1995 as an adjuvant treatment following surgical resection of primary colorectal tumors [13, 14]. Subsequent larger studies, however, showed edrecolomab to be inferior to established chemotherapy, leading to the withdrawal of its market authorization. Since then, several different immunotherapeutic approaches targeting EpCAM have been developed by utilizing monoclonal antibodies [10, 15], bispecific (trifunctional) antibodies [16, 17], or conjugates with either toxins [18] or Interleukin 2 (IL-2) [19]. The majority of these antibody drug candidates have entered clinical trials for cancer treatment [20], while Catumaxomab (trade name Removab), a trifunctional bispecific mAb [16, 21], was approved in the European Union (EU) in April 2009 for intraperitoneal (i.p.) treatment of malignant ascites (MA) in patients with EpCAM-positive carcinomas. Moreover, subsequent data from clinical trials of other anti-EpCAM antibody-based drug candidates, such as Edrecolomab [9] and Adecatumumab (MT201) [10, 22], suggested that anti-EpCAM monoclonal antibodies have only limited anti-tumor effects, primarily through activation of complement-dependent cytotoxicity (CDC) and antibody-dependent cellular cytotoxicity (ADCC) [22, 23]. Adecatumumab (MT201), a fully human IgG1 monoclonal antibody targeting EpCAM, has cancer cell-killing activity that is independent of K-Ras status [24]. A phase II study in patients with metastatic breast cancer confirmed the overall safety and feasibility of single-agent treatment with Adecatumumab [10]. At the time of writing, the exact roles of EpCAM in carcinogenesis and malignant progression have yet to be elucidated, and the low efficacy of current anti-EpCAM drug candidates in clinical trials highlights a need for the development of more efficacious anti-EpCAM antibodies.

EpCAM, a polypeptide of 314 amino acids (aa), contains an extracellular domain (EpEX) of 242 aa, a transmembrane domain of 23 aa, and an intracellular domain (EpICD) of 26 aa [25]. EpEX, which closely resembles the fourth and fifth EGF-like motifs involved in cell-matrix adhesion, is composed of two epidermal growth factor-like domains (aa 27–59 and 66–135) and a cysteine-poor region, while EpICD is a short sequence [26]. However, the second motif does not represent an EGF-like repeat, and instead resembles a thyroglobulin (TY) type repeat [27–29]. TY type 1 domains are conserved in a number of proteins and are capable of binding, thereby inhibiting certain cathepsins (cysteine proteases) involved in cancer progression [30, 31]. Whether EpCAM acts as a substrate or inhibitor of cathepsins is not known. EpEX and EpICD are separated through intramembrane proteolysis (RIP), a process that is activated by TACE/ADAM17, a γ-secretase complex containing presenilin 2 (PS-2) [32] and α-, β-secretase [33]. Recent studies have shown that nuclear translocation of EpICD allows it to function as a signaling transducer, suggesting an important role for proteolytic cleavage of EpCAM into EpICD and EpEX in EpCAM-mediated malignant progression [32, 34]. Accumulation of EpICD in the nucleus has been found to be associated with tumor malignancy [34] and with undifferentiated embryonic stem cells (ESCs) [35]. However, the exact mechanisms by which EpCAM cleavage and EpEX signaling lead to tumor malignancy are yet to be established.

EpCAM expression has been detected in certain tumor initiation cells (TICs) [36, 37], suggesting EpCAM as a possible target for enrichment of TICs and circulating tumor cells (CTCs) [38–41]. TICs are considered to have greater drug resistance and metastatic potential than non-TICs [42, 43]. Numerous studies have also confirmed that TICs are present within a broad spectrum of cancer types, and that TICs have tumorigenic potential [44, 45]. However, in the absence of an effective biomarker with high specificity, it is difficult to elucidate the molecular mechanisms underlying TIC development, and to identify an appropriate therapy against TICs. Hence, there remains an urgent need for the development of novel therapeutics against TICs/CTCs.

In this study, we generated five mAbs targeting EpCAM, including EpAb2-6, which demonstrated a unique capability to directly induce apoptosis in cancer cells and to inhibit EpICD cleavage. This mAb is a potential therapeutic candidate for treatment of CRC and pancreatic cancer.

## RESULTS

### Generation and characterization of mAbs recognizing EpCAM

**Figure 1:**
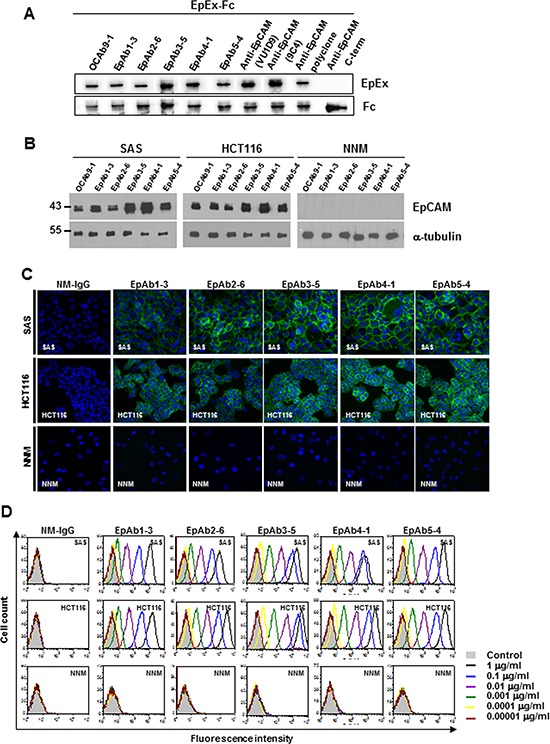
Characterization of anti-EpCAM mAbs (EpAb1-3, EpAb2-6, EpAb3-5, EpAb4-1, and EpAb5-4) Binding activities of anti-EpCAM mAbs were measured by Western blotting **A.** and **B.** immunofluorescent staining **C.** and flow cytometry **D.**

**Figure 2:**
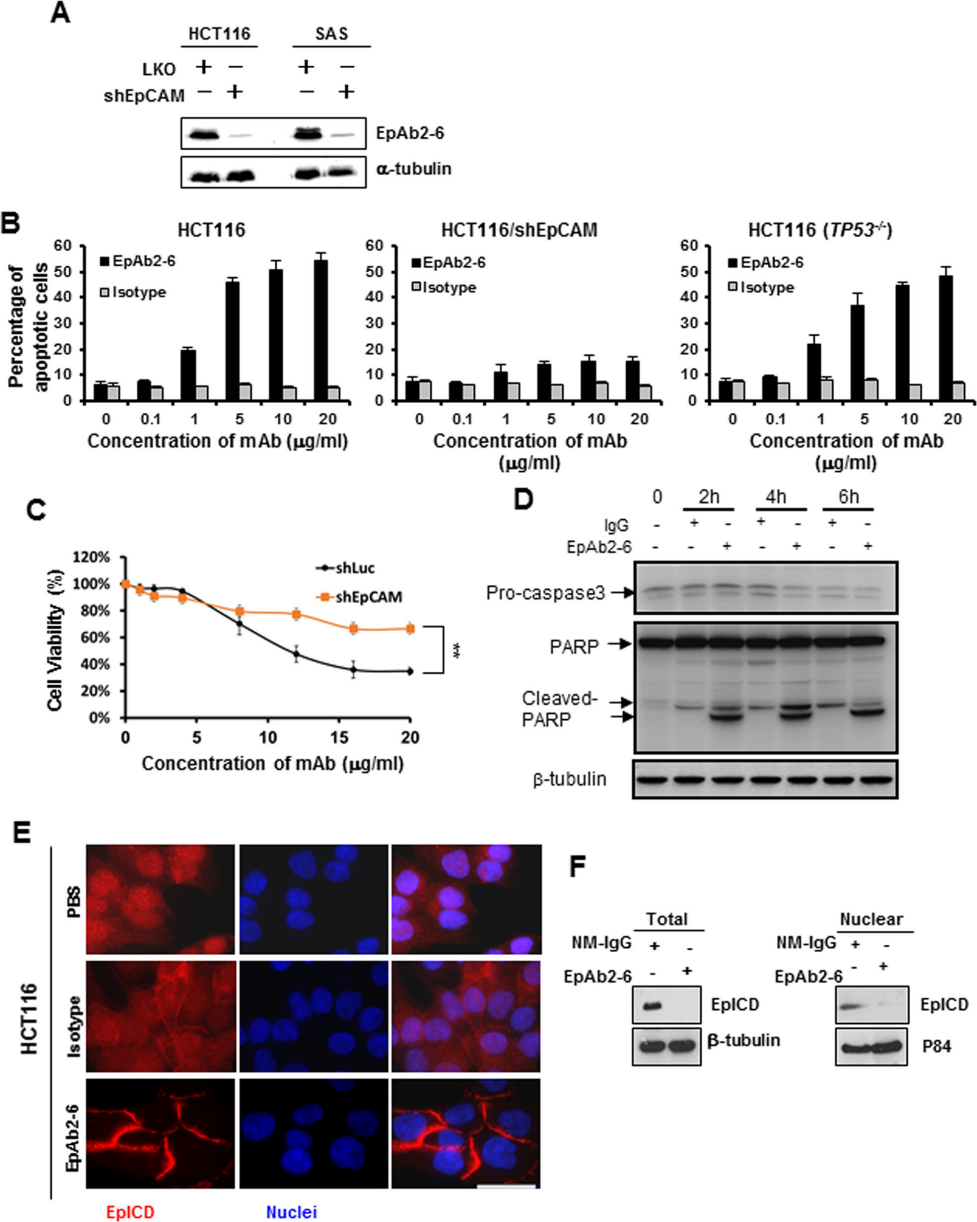
Inhibition of cancer cell growth *in vitro* by EpAb2-6 HCT116 and SAS cells were transfected with *EpCAM* shRNA plasmids (shEpCAM). **A.** Western blot analyses were performed to evaluate EpAb2-6 binding to *EpCAM*-knockdown HCT116, SAS, and mock cells. **B.** HCT116, HCT116/shEpCAM, and HCT116 (*TP53^−/−^*) cells were treated with EpAb2-6 (0–20 μg/ml) or isotype control (mouse myeloma IgG2a) for 6 h, and cell death was measured by flow cytometry with Annexin V-FITC and PI double staining. Annexin V-FITC was used to determine the percentage of cells within the population that were actively undergoing apoptosis at an early stage (6 hours). Propidium iodide (PI) was used to distinguish between viable and nonviable cells. **C.** EpCAM knockdown inhibits EpAb2-6, which induces repression of cell viability. HCT116 cells stably expressing control shRNA (shLuc) or shEpCAM were treated with EpAb2-6 (0 – 20 μg/ml) for 48 h. Error bars show mean ± SD (Student's *t*-test, **p < 0.01). **D.** Non-attachment assay. HCT116 cells were incubated under Non-attachment conditions with EpAb2-6, which increased cleavage of capase-3 and PARP. **E.** EpAb2-6 inhibits EpICD cleavage and nuclear localization. Immunofluorescence images of EpICD cellular localization in HCT116 cells treated with EpAb2-6 (40 μg/ml), isotype control (mouse IgG2a, 40 μg/ml), or PBS for 48 hours. (Bar = 10 μm.) **F.** EpAb2-6 inhibits EpICD production and nuclear translocation. HCT116 cell lines transfected with EpCAM-v5 plasmids were treated with either NM-IgG or EpAb2-6 (40 μg/ml) for 48 hours, and the lysates were subsequently subjected to Western blotting.

We recently established a highly specific mAb, OCAb9-1, against the cell surface protein EpCAM, and found that EpCAM was highly expressed in SAS and HCT116 cells. OCAb9-1 also specifically recognized recombinant human EpCAM/Fc chimera (960-EP, R&D Systems, Minneapolis, Minn, USA) (Fig. 1A) and several human cancer cells, but not normal cells. OCAb9-1 was unable to induce cancer cell apoptosis; we therefore attempted to develop pro-apoptotic antibodies by generating a monoclonal antibody against EpCAM. From more than 3,000 hybridoma clones, we identified 49 anti-EpCAM mAbs, five of which possessed high binding activity to several human cancer cell lines (SAS, NPC, HCT116, H441, MCF7, BxPC-3, and SKOV-3), but not normal cell lines (HUVECs and NNM). The binding affinities of these five anti-EpCAM mAbs are summarized in Table 1A. Closer examination by Western blotting (Fig. 1B), immunofluorescent analysis (Fig. 1C), and flow cytometry analyses (FACS) (Fig. 1D) revealed that these mAbs exhibited extremely high cell surface binding activity to HCT116 and SAS cells, without showing any binding activity to NNM cells. All of these mAbs have very high affinity to EpCAM, with kinetic constants ranging from 10^−9^ ∼ 10^−13^ (Table 1B). Western blotting (Fig. 2A) revealed a dramatic decrease in EpAb2-6 after EpCAM knockdown, thereby confirming the specificity of EpAb2-6 against EpCAM.

**Table 1a:** Summary of the main features of anti-EpCAM mAbs

					Cell lines									
mAb clone	ELISA	WB	Flow	Isotype	SAS	NPC	H441	H1993	HCT116	SKOV-3	MCF7	BxPC-3	HUVECs	NNM
EpAb1-3	+	+	+	IgG1,κ	+ +	+	+	+	+	+/−	+	+	−	−
EpAb2-6	+	+	+	IgG2a, κ	+ +	+	+	+	+	+/−	+	+ +	−	−
EpAb3-5	+	+	+	IgG2b, κ	+ + +	+ +	+ +	+ +	+ +	+ +	+ +	+ +	−	−
EpAb4-1	+	+	+	IgG1, κ	+ +	+	+ +	+	+	+	+	+ +	−	−
EpAb5-4	+	+	+	IgG1, κ	+ +	+	+	+	+	+/−	+	+	−	−

Key: +, indicates binding (+ + +, OD 490 nm >1.5; + +, OD 490 nm 1–1.5; +, OD 490 nm 0.5–1; +/−, OD 490 nm 0.2–0.5); indicates no binding (OD 490 nm <0.2 ); WB, Western blot; Flow, flow cytometry.

**Table 1b:** Kinetic constants and binding affinities of anti-EpCAM mAbs

mAb colon	K_d_(M)	K_on_(M^−1^S^−1^)	K_off_(S^−1^)
EpAb1-3	1.833 × 10^−9^	1.849 × 10^5^	3.389 × 10^−4^
EpAb2-6	3.491 × 10^−10^	4.007 × 10^5^	1.399 × 10^−4^
EpAb3-5	≤ 4.66 × 10^−13^	2.961 × 10^6^	1.38 × 10^−6^
EpAb4-1	1.228 × 10^−12^	2.865 × 10^5^	3.519 × 10^−7^
EpAb5-4	2.431 × 10^−10^	6.221 × 10^5^	1.513 × 10^−4^
hEpAb2-6	6.773 × 10^−10^	3.756 × 10^5^	2.544 × 10^−4^

K_on_ and K_off_ were measured by SRP in a BIAcore using purified mAb, and the K_d_ was calculated by BIAevaluation software.

### The EpAb2-6 antibody inhibits the growth of cancer cells

There are currently no published reports of an anti-EpCAM antibody that can directly induce apoptosis; here, we examined whether our newly generated anti-EpCAM mAbs possessed this ability. Of the five novel mAbs (Supplementary Fig. S1), EpAb2-6 was able to induce apoptosis of SAS, SW620, HCT116, and HCT116 (*TP53^−/−^*), but not HCT116/shEpCAM or normal cell lines (NNM) (Fig. 2A and 2B, Table 2 and Supplementary Figs. S2, S3A and S3B). Specifically, the percentage of cell viability in stable clones expressing a firefly luciferase shRNA (shLuc) was reduced to 34% after EpAb2-6 (20 μg/ml) treatment for 48 hours. In contrast, EpAb2-6 treatment of EpCAM knockdown cells resulted in a smaller decrease (66% cell viability), illustrating the inhibitory effect of EpAb2-6 on cell growth (Fig. 2C). On the other hand, we found that EpCAM knockdown decreased the viability of untreated cells (data not shown), demonstrating that loss of EpCAM has negative effects on cell survival in HCT116. In the non-attachment cell death assay, HCT116 cells were treated with the same dosage of EpAb2-6 for 2, 4, and 6 h, followed by Western blotting analysis of PARP and caspase-3 cleavage. It was apparent that EpAb2-6 treatment led to an increase in PARP cleavage levels and a decrease in pro-caspase-3, compared to control IgG (Fig. 2D). We previously reported that triggering EpICD cleavage and its subsequent nuclear translocation are involved in cancer initiation in TICs [34]. To determine whether EpAb2-6 inhibits EpICD cleavage and its subsequent nuclear translocation, we examined the localization of EpICD following treatment of HCT116 cells with EpAb2-6; we report that EpICD localized to the membrane-bound region of EpAb2-6-treated cells (Fig. 2E). Soluble (cleaved) EpICD was observed in both the cytoplasm and the nucleus of HCT116 cells treated with isotype control antibody (Fig. 2E). The presence of soluble EpICD in HCT116 cells was reduced after EpAb2-6 treatment, as shown by Western blot analysis (Fig. 2F). Collectively, these results suggest that EpAb2-6 may induce cancer cell cytotoxic activity by inducing an apoptosis pathway, and that EpAb2-6 inhibits EpICD nuclear translocation by blocking the cleavage of EpCAM.

**Table 2:** Apoptosis effect of EpAb2-6 in cancerous or normal cells

Cancer type (cell line)	Antibody	Concentration tested (μg/ml)					
		0	0.1	1	5	10	20
Oral cancer(SAS)	EpAb2-6	13.84	20.91	27.56	40.56	43.89	50.4
	NM-IgG	14.82	13.08	14.05	12.24	10.93	10.99
Colon cancer(HCT116 (TP53^+/+^))	EpAb2-6	11.1	14.12	30.82	42.18	51.7	52.26
	NM-IgG	12.5	8.82	8.08	12.06	10.22	9.54
Colon cancer(HCT116 (TP53^−/−^))	EpAb2-6	9.45	25.81	32.67	40.77	56.66	55.5
	NM-IgG	12.82	11.97	12.99	17.14	13.69	13.95
Normal nasal mucosal epithelia(NNM)	EpAb2-6	9.54	9.12	8.49	9.55	8.77	11.5
	NM-IgG	10.69	9.51	8.21	9.56	9.4	10.2

### Identification of B cell epitopes of EpAb2-6

**Figure 3:**
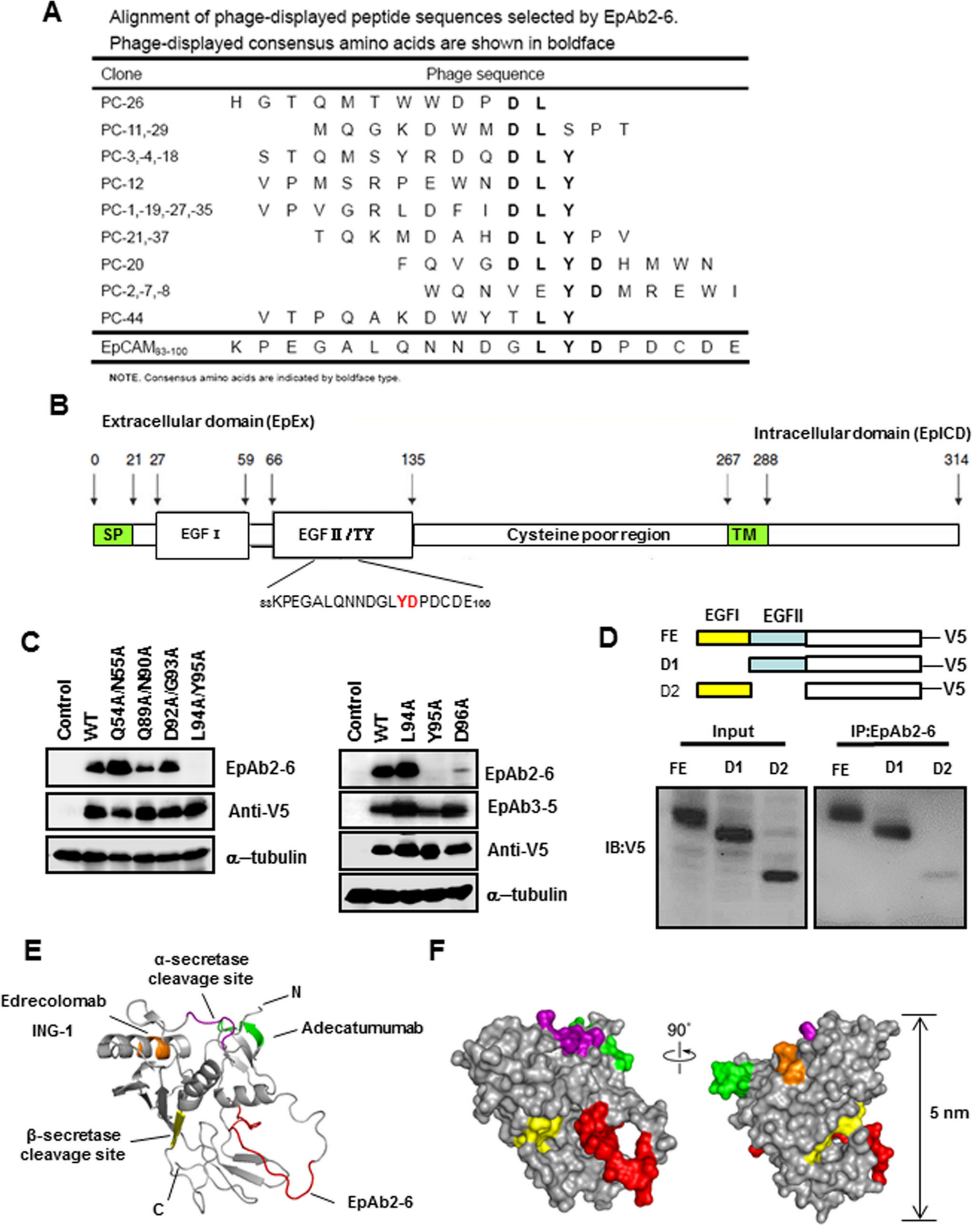
Identification of the B cell epitope of EpAb2-6 **A.** Alignment of phage-displayed peptide sequences selected by EpAb2-6. **B.** EpCAM mutations with amino acid substitutions in the EGF-I (Q54A/N55A) or EGF-II domain (Q89A/N90A, D92A/G93A, L94A/Y95A, L94A, Y95A, or D96A). **C.** The indicated EpCAM mutants were expressed in HEK293 cells. Cellular protein extracts were subjected to Western blot analysis using EpAb2-6 and EpAb3-5 antibodies. Substitutions of Y95 and D96 reduced EpAb2-6 binding activity. **D.** Various EpCAM constructs with different EGF-domains of EpAB2-6 binding sites (FE: full length EpCAM; D1: EGF-I domain deletion; D2: EGF-II/TY domain deletion) are shown. These constructs were transiently transfected into HEK293 cells to evaluate their binding ability with EpAb2-6. Epitopes of anti-EpCAM antibodies are mapped to a structural model of EpEx. **E.** A ribbon diagram representation of the complete EpEx structure. The epitope of Edrecolomab (mouse Ab) and ING-1 (humanized Ab) is shown in orange. The epitopes of Adecatumumab (MT201; human Ab) and EpAb2-6 are colored green and red, respectively. The cleavage sites of α-secretase (Adam) and β-secretase (BACE1) are colored purple and yellow, respectively. N and C indicate the N and C terminus of EpEx, respectively. **F.** The molecular surface of EpEx is color coded as described in E.

To identify the binding motif of the EpAb2-6 antibody, we sequenced DNA from 18 phage clones that were highly reactive with this antibody, but less reactive with normal mouse IgG. All clones were found to contain 36 nucleotides (therefore encoding 12 amino acids). Peptide sequences were aligned using MacDNAsis software, which revealed that the phage-displayed LYD motif corresponds to amino acid residues at positions 94–96 of EpCAM (Fig. 3A). To further confirm that EpAb2-6 recognized the LYD motif in EpCAM, we constructed cDNA sequences encoding the first (aa 27-59; EGF-I domain) and second (aa 66-135; EGF-II/TY domain) EGF-like repeat of human EpCAM. PCR-based site-directed mutagenesis was subsequently used to introduce mutations into these domains (Fig. 3B). Western blotting was used to determine the reactivity of EpAb2-6 or EpAb3-5 antibodies towards these EpCAM mutants (Fig. 3C). Amino acid mutations at positions Y95 or D96 in the EGF-II domain of EpCAM caused marked reductions in binding activity of EpAb2-6, but not EpAb3-5 (Fig. 3C). However, amino acid mutations at positions Q54, N55, Q89, N90, D92, G93, and L94 had no effect on the binding affinity of EpAb2-6 to EpCAM (Fig. 3C). We also established different EGF-like domain deletion clones D1 (EGF-I domain deletion) and D2 (EGF-II/TY domain deletion) for immunoprecipitation. Western blotting was used to demonstrate that EpAB2-6 does not bind clone D2 (Fig. 3D). Most importantly, these lines of evidence confirm that EpAb2-6 binds to the EGF-II/TY domain instead of the EGF-I domain. To further elucidate the interaction of EpAb2-6 with EpCAM, we built a molecular model to mimic the extracellular portion of EpCAM (EpEx) based on previously reported crystal structural information [51]. We also labeled the binding epitopes of anti-EpCAM antibodies and the cleavage sites of secretases [33, 52] (Figs. 3E and 3F). The ribbon and surface models show that the binding epitope of EpAb2-6 is different to that of the three anti-EpCAM antibodies currently in clinical trials; i.e., edrecolomab, ING-1, and adecatumumab. Interestingly, we found that the epitope of EpAb2-6 is localized in the TY loop and is very close to the cleavage site of β-secretase BACE1 (Beta-site APP Cleaving Enzyme) (22 Å). It was recently demonstrated that the release of EpICD from EpCAM triggers proliferation- and stemness-enhancing signaling in cancer cells [32, 34]. Moreover, the TY loop has been reported to be critical for stabilizing cis-dimer architecture of two EpCAM molecules, which mediate cell-to-cell contact [51]. We therefore hypothesize that the binding of EpAb2-6 may lead to steric hindrance that disrupts cis-dimer formation of EpCAM and inhibits cleavage of EpEx by β-secretase. This in turn compromises the release of EpICD, eventually leading to cancer cell apoptosis.

### *In vivo* tumor targeting of anti-EpCAM mAbs

**Figure 4:**
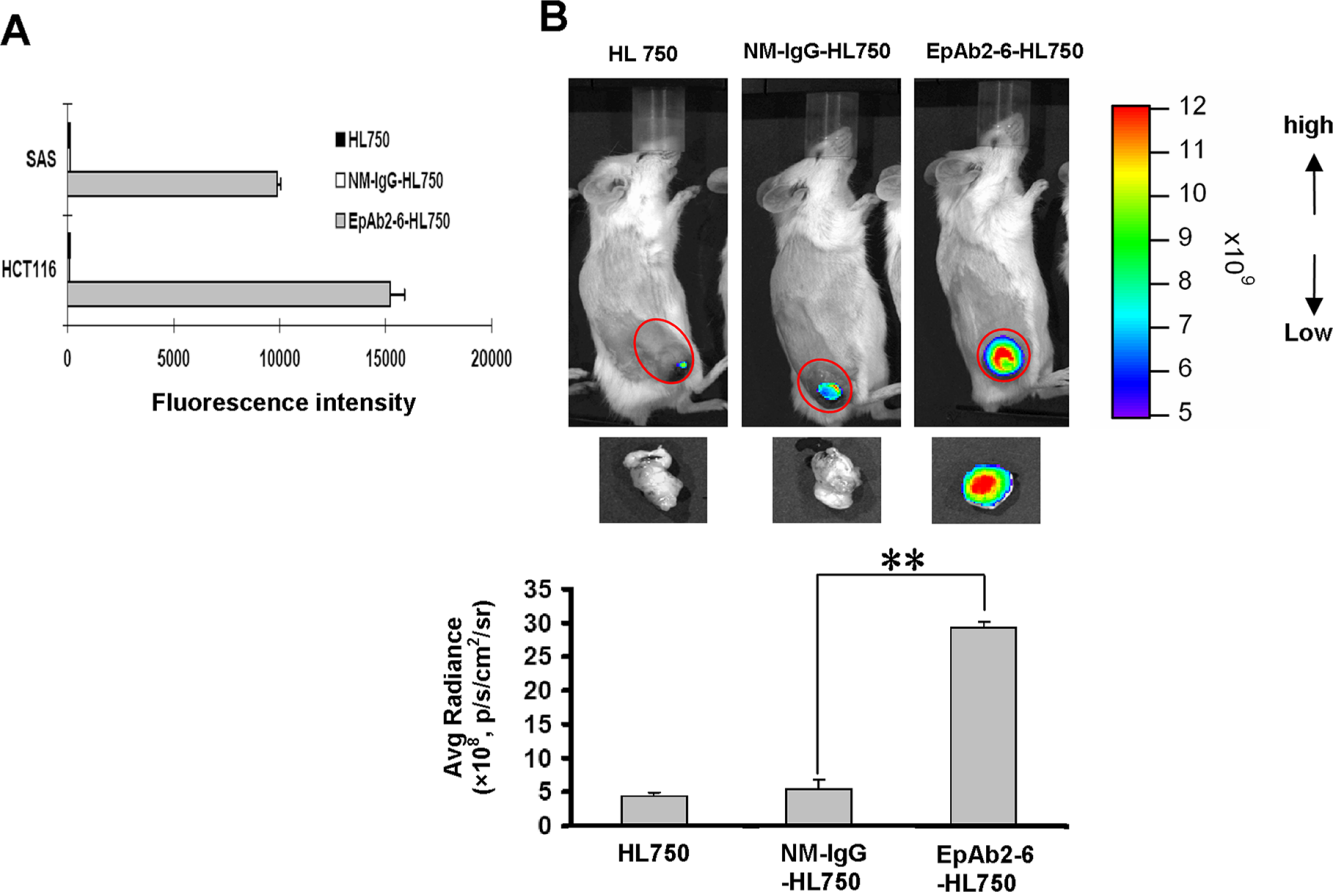
Tumor-homing ability of anti-EpCAM mAb in human colon cancer xenografts **A.** The expression level of EpCAM on cancer cell surfaces was determined by flow cytometry analysis using EpAb2-6-HL750. HL750 and NM-IgG-HL750 were used as controls. **B.** *In vivo* imaging of SCID mice bearing HCT116 human colon tumor xenografts was performed after intravenous injection of EpAb2-6-HL750, NM-IgG-HL750, or HL750. NIR fluorescence images were acquired at 48 hours post-injection (top). Red circles indicate the tumor loci. The signal intensity of the tumor area was quantified using IVIS software. Tumor distributions of EpAb2-6-HL750, NM-IgG-HL750, and HL750 at 72 hours post-injection are shown. Signal intensities for the tumor and organs were measured using IVIS software. Error bars show mean ± SD (*n* = 3) (Student's *t*-test, **p < 0.01) (below).

Site-directed conjugation was used to specifically couple antibodies to HiLyte-750 acid NHS ester via the NHS functional group, thereby producing HiLyte-750 conjugated EpAb2-6 (EpAb2-6-HL750) or HiLyte-750 conjugated normal mouse-IgG (NM-IgG-HL750). *In vitro* analysis indicated that EpAb2-6-HL750 can bind to SAS and HCT116 cells with high affinity (Fig. 4A). To determine the suitability of EpAb2-6 for use in tumor imaging assays, we injected 5 nM EpAb2-6-HL750 and control (NM-IgG-HL750 or HiLyte Fluor™ 750 dye only) into mice bearing HCT116-derived colon tumor xenografts. Mice and tissues were imaged using a Xenogen IVIS 200 imaging system (Excitation: 710/760 nm; Emission: 810/875 nm) at the indicated times. At 48 hours after injection, the near-infrared (NIR) fluorescence signal intensity in the tumor tissues of EpAb2-6-HL750-treated mice was significantly higher than that of mice treated with non-conjugated HL750 and NM-IgG-HL750 (Fig. 4B). We subsequently sacrificed and anatomized the mice to investigate the tissue distribution of EpAb2-6. Tumor tissues exhibited strong and selective accumulation of EpAb2-6-HL750, which was 6.50- and 5.32-fold higher than that of non-conjugated HL750- and NM-IgG-HL750-treated mice, respectively (Fig. 4B). These results indicate that EpAb2-6-HL750 exhibits high levels of tumor binding.

### Combinatorial treatment of human colon carcinoma xenografts with EpAb2-6 and IFL

**Figure 5:**
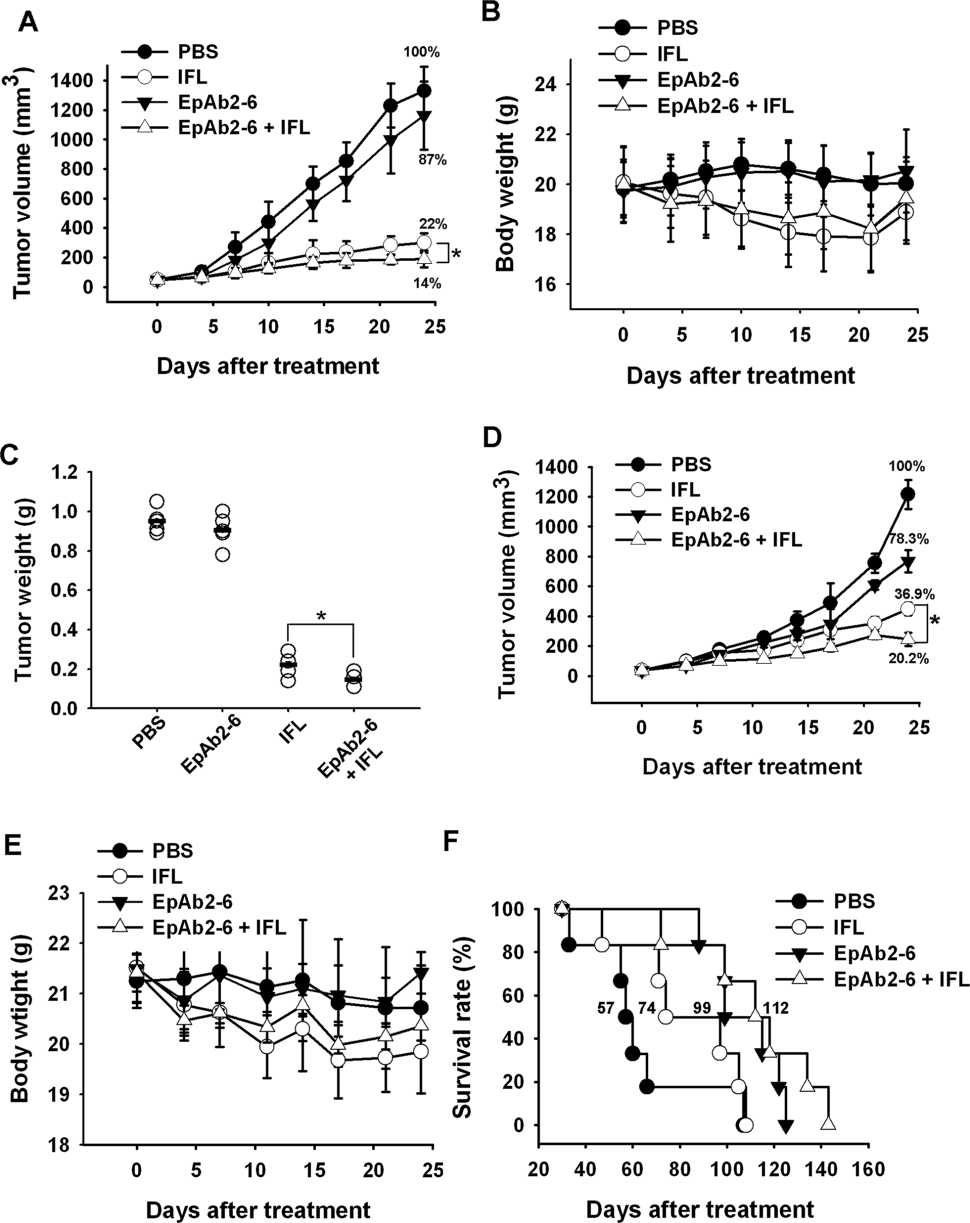
Effect of combinatorial treatment with EpAb2-6 and IFL on mice bearing HCT116 tumors **A, D.** Mice bearing HCT116-derived tumor xenografts were treated with EpAb2-6, IFL, EpAb2-6 in combination with IFL, or PBS. The sizes of tumors in each group were determined on the indicated days. Error bars show mean ± SD (*n* = 6) (Student's *t*-test, *p < 0.05). **B, E.** Average body weight of each group is shown on the indicated days. Error bars show mean ± SD. **C.** Tumor weight from (A) was measured at the end of the treatment period. (Student's *t*-test, *p < 0.05.) **F.** A Kaplan-Meier survival curve from **(D)** indicates that mice bearing xenografts treated with EpAb2-6 or EpAb2-6 in combination with IFL had a greater survival rate than those treated with IFL or PBS (*n* = 6).

Since EpCAM knockdown and EpAb2-6 treatment disrupted cancer cell growth and induced cancer cell apoptosis *in vitro*, we investigated whether EpAb2-6 could be used to directly inhibit tumor growth *in vivo*. EpAb2-6 was shown to be able to inhibit the growth of human oral and lung cancer (data not shown) in tumor-bearing mice. Targeted therapies, such as bevacizumab, cetuximab, and panitumumab, in combination with chemotherapy are more effective than chemotherapy alone [53, 54]. Hence, there is interest in developing targeted therapies based on EpAb2-6 for use in combination with IFL (irinotecan, leucovorin, and fluorouracil). Mice with colon cancer xenografts treated with a combination of EpAb2-6 and IFL exhibited smaller tumors than those in mice treated with IFL alone (*p < 0.05); the tumors of the IFL group gradually increased in size, becoming 1.6-fold larger than the tumors of the EpAb2-6 + IFL group by day 25 (Figs. 5A and 5D). Body weight was not significantly different between the two treatment groups (Fig. 5B and 5E). By the end of the treatment period, the average tumor weight in mice treated with IFL was 0.23 g, compared to 0.146 g in mice treated with EpAb2-6 + IFL and 0.952 g in mice injected with PBS (Fig. 5C).

To further confirm that EpAb2-6 increases therapeutic efficacy against colon cancer, we compared the survival rates of tumor-bearing mice under different treatment regimens. The median overall survival rates for tumor-bearing mice after treatment with PBS, IFL, EpAb2-6, and EpAb2-6 + IFL were 57, 74, 99, and 112 days, respectively (Fig. 5F). The Kaplan-Meier curve for overall survival (OS) of the PBS group was significantly different to those of the EpAb2-6 and EpAb2-6 + IFL groups (log rank test *p* = 0.0493 and *p* = 0.0271, respectively). However, no significant difference in OS was observed between the IFL and EpAb2-6 + IFL groups (log rank test *p* = 0.0972). Additionally, we have used another cancer cell line, SW620, to further demonstrate the therapeutic potential of EpAb2-6 through a double-blind experiment. SW620 is p53 mutation and less sensitive to IFL therapy [55]. The results confirmed our previous finding in that EpAb2-6 in combination with IFL had higher therapeutic efficacy at reducing tumor growth than IFL alone (Supplementary Fig. S3C and 3D).

### EpAb2-6 increases the survival rate of mice in colon and pancreatic cancer metastatic animal models

**Figure 6:**
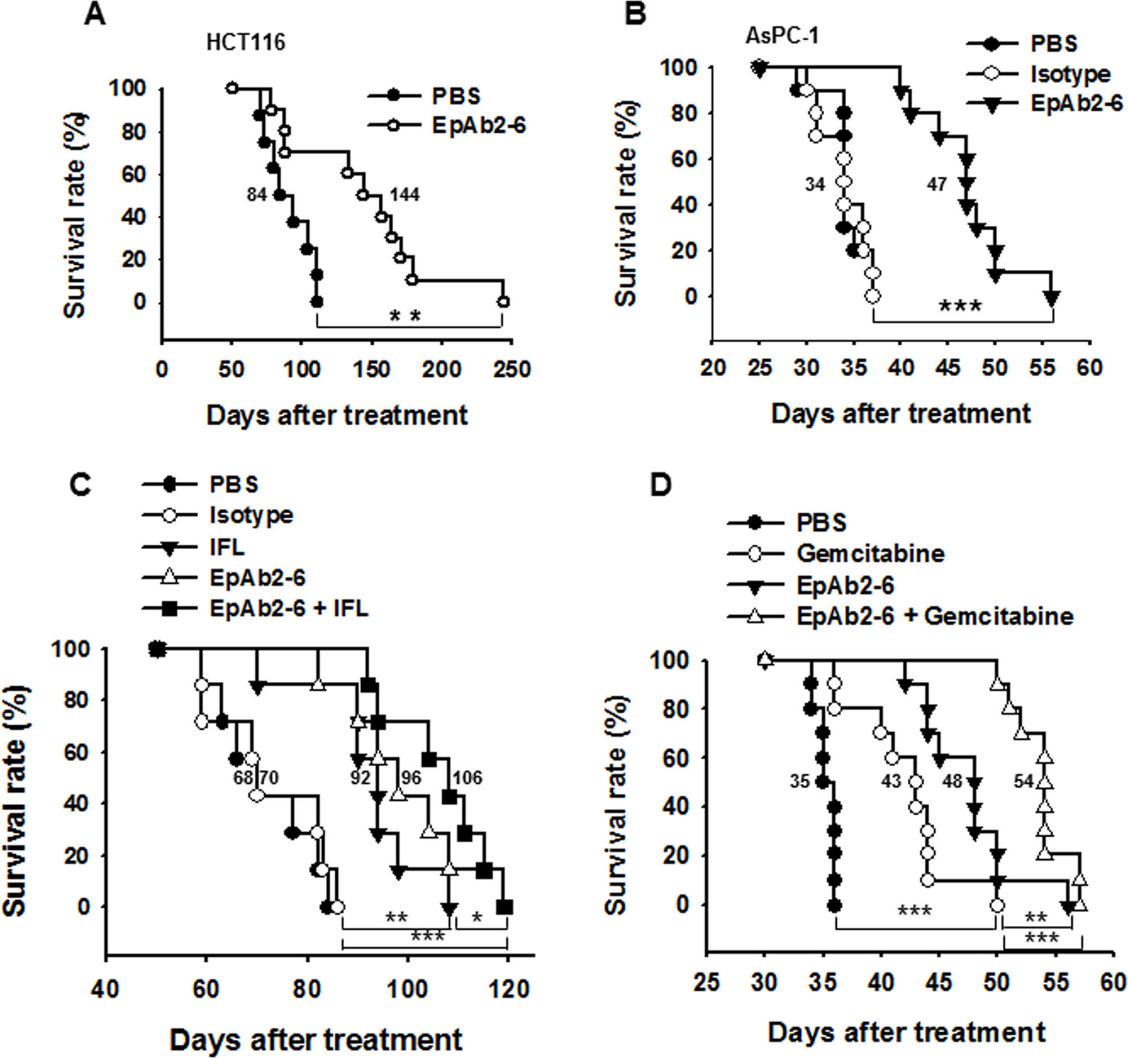
EpAb2-6 enhances survival in an animal model of tumor metastasis **A.** NOD/SCID mice were intravenously injected with 1 × 10^6^ HCT116 cells, and then were treated with either PBS or EpAb2-6 (*n* = 10). The survival curves indicate that mice treated with EpAb2-6 exhibited a greater survival rate than those treated with PBS. **B.** NOD/SCID mice were intravenously injected with 1 × 10^6^ AsPC-1 cells, and were then treated with either PBS, isotype control (Myeloma IgG2a), or EpAb2-6 (*n* = 10). The survival curves indicate that mice treated with EpAb2-6 exhibited a greater survival rate than those treated with Isotype. **C.** Mice bearing metastatic HCT116-derived tumors were treated with EpAb2-6, Isotype, IFL, EpAb2-6 in combination with IFL, or PBS. Kaplan-Meier survival curves indicate that mice bearing metastatic cancer cells treated with EpAb2-6 or EpAb2-6 in combination with IFL had a greater survival rate than mice treated with IFL alone or PBS (*n* = 7) (Log rank test, **p < 0.01, ***p < 0.001). **D.** Mice bearing metastatic AsPC-1-derived tumors were treated with EpAb2-6, Gemcitabine, EpAb2-6 in combination with Gemcitabine, or PBS. Kaplan-Meier survival curves indicate that mice bearing metastatic cancer cells treated with EpAb2-6 or EpAb2-6 in combination with Gemcitabine had a greater survival rate than mice treated with Gemcitabine alone or PBS (*n* = 10) (Log rank test, **p < 0.01, ***p < 0.001).

Aggressive tumors with rapid growth often metastasize by invading the surrounding tissue, and they are always associated with poor prognosis. We used a colon carcinoma metastatic animal model to investigate whether EpAb2-6 treatment could increase the median overall survival of metastatic tumor-bearing mice. NOD/SCID mice were injected intravenously with HCT116 cells; mice bearing circulating HCT116 cells were intravenously treated with EpAb2-6 or an equivalent volume of PBS at 24 and 96 hours after cell injection (antibody was delivered at 20 mg/kg/dose, for a total dose of 40 mg/kg). The median overall survival of tumor-bearing mice treated with EpAb2-6 (144 days) was significantly higher than that of PBS-treated mice (84 days; Fig. 6A). The difference in overall survival (OS) between the PBS and EpAb2-6 treatment groups (as determined using Kaplan-Meier curves) was found to be statistically significant (log rank test *p* = 0.0078). To further verify the therapeutic efficacy of EpAb2-6, we treated colon carcinoma metastatic animal models with a combination of EpAb2-6 and IFL. The survival rate of mice treated with a combination of EpAb2-6 and IFL was found to be higher than that of mice treated with IFL alone. The median overall survival rates of tumor-bearing mice after treatment with PBS, Isotype, IFL, EpAb2-6, and EpAb2-6 + IFL were 68, 70, 92, 96, and 106 days, respectively (Fig. 6C). Kaplan-Meier curves for overall survival (OS) were significantly different between groups treated with either IFL alone or EpAb2-6 + IFL (log rank test *p* = 0.017) (Fig. 6C).

Pancreatic ductal adenocarcinoma (PDA) is among the most intractable of human malignancies, with an overall 5-year survival rate of only 5–6% [56]. Therefore, new approaches for the development of more effective treatments for pancreatic cancer are desperately needed. The EpCAM overexpression rate in pancreatic primary tumors ranges between 33% and 60% [57, 58]. Moreover, EpAb2-6 specifically recognizes several human pancreatic cancer cells: AsPC-1, BxPC-3, and PANC-1 (Supplementary Fig. S4A). We found that EpAb2-6 not only induces AsPC-1 cancer cell apoptosis (Supplementary Fig. S4B), but also increases the median overall survival of metastatic tumor-bearing mice (Fig. 6). NOD/SCID mice were injected intravenously with AsPC-1 cells. The median overall survival of tumor-bearing mice treated with EpAb2-6 was significantly higher than that of PBS and isotype-treated mice (Fig. 6B). The median overall survival times of AsPC-1-derived tumor-bearing mice after treatment with PBS, isotype control IgG2a, and EpAb2-6 were 34, 34, and 47 days, respectively (Fig. 6B). The difference in overall survival (OS) between the isotype control IgG2a and EpAb2-6 treatment groups (as determined using Kaplan-Meier curves) was found to be statistically significant (log rank test *p* < 0.001).

PDA is among the most lethal human cancers, in part because it is insensitive to many chemotherapeutic drugs. Decades of studies have borne witness to the failure of many chemotherapeutic regimens, and the current standard-of-care therapy, gemcitabine, extends patient survival by only a few weeks [59, 60]. To further verify the therapeutic efficacy of EpAb2-6, we treated pancreatic carcinoma metastatic animal models with a combination of EpAb2-6 and gemcitabine. The survival rate of mice treated with a combination of EpAb2-6 and gemcitabine was found to be higher than that of mice treated with gemcitabine alone. The median overall survival rates of tumor-bearing mice after treatment with PBS, gemcitabine, EpAb2-6, and EpAb2-6 + gemcitabine were 35, 43, 48, and 54 days, respectively (Fig. 6D). The Kaplan-Meier curve for overall survival (OS) of the group treated with gemcitabine alone was significantly different to those of the EpAb2-6 and EpAb2-6 + gemcitabine groups (log rank test *p* = 0.0085 and *p* < 0.001, respectively) (Fig. 6D). These results demonstrate that EpAb2-6 increases the therapeutic efficacy of gemcitabine against PDA.

### Inhibition of cancer cell growth by humanized EpAb2-6 (hEpAb2-6) antibody

**Figure 7:**
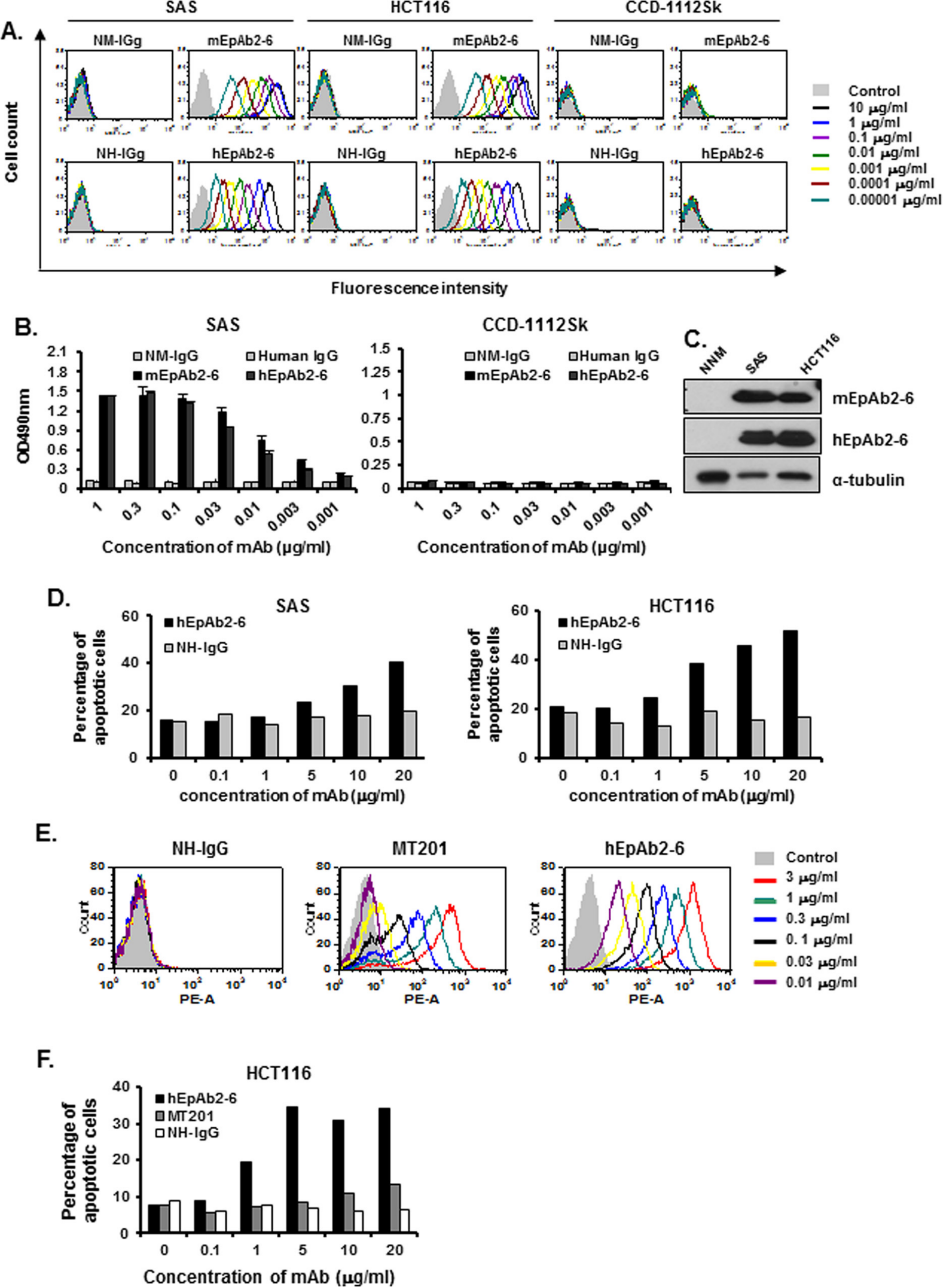
Development of a humanized antibody against EpCAM The CDRs of EpAb2-6 were grafted onto a human IgG1 backbone to create humanized EpAb2-6 (hEpAb2-6). The binding activity of hEpAb2-6 to human cancer cell lines is shown. Flow cytometry analysis **A.** and ELISA **B.** were performed to measure the binding activity of mEpAb2-6 and hEpAb2-6 to SAS, HCT116, and CCD-1112Sk cells. Normal mouse IgG (NM-IgG) and normal human IgG (human IgG) were used as negative controls. **C.** Western blot analyses of mEpAb2-6 and hEpAb2-6 against NNM, SAS, and HCT116 cells. **D.** SAS and HCT116 cells were treated with hEpAb2-6 (0–20 μg/ml) for 6 h, and cell death was measured by flow cytometry with Annexin-V FITC and PI double staining. Binding activity of hEpAb2-6 and MT201 to human cancer cell lines. Flow cytometry analysis **E.** was performed to measure the binding activity of hEpAb2-6 and MT201 to HCT116 cells. Normal human IgG (NH-IgG) was used as a negative control. **F.** HCT116 cells were treated with hEpAb2-6 and MT201 (0–20 μg/ml) for 6 h, and cell death was measured by flow cytometry with Annexin-V FITC and PI double staining.

EpAb2-6 exhibits high affinity and potent activity for induction of cancer cell apoptosis, which suggests it may have potential as a therapeutic antibody. Murine mAbs have been shown to be of limited clinical use because of their short serum half-life, inability to trigger human effector functions, and the observation that they induce a human anti-murine antibody (HAMA) response (LoBuglio et al., 1989). To develop humanized mAbs, we sequenced the V_H_ and V_L_ segments of EpAb2-6 from hybridoma cell lines. The CDRs of EpAb2-6 were grafted onto a human IgG1 backbone to create humanized EpAb2-6 (hEpAb2-6). The hEpAb2-6 construct was expressed in CHO-K1 cells and purified from culture supernatants. The hEpAb2-6 antibody, which maintained the specificity of murine EpAb2-6 (mEpAb2-6), recognized both SAS and HCT116 cancer cells, but not CCD-1112Sk normal cells (Fig. 7A). Cellular ELISA and Western blotting further demonstrated that hEpAb2-6 possessed high binding activities (Fig. 7B and 7C). The affinities of EpAb2-6 and hEpAb2-6 for EpCAM were analyzed by surface plasmon resonance, and shown to be 0.3491 nM and 0.6773 nM, respectively (Table 1b). Furthermore, *in vitro* studies using SAS and HCT116 cell lines revealed that hEpAb2-6 induced cancer cell apoptosis (Fig. 7D). Comparison of hEpAb2-6 generated in our lab with MT201 indicated that hEpAb2-6 has (i) much higher binding affinity (Fig. 7E) and (ii) a greater ability to induce direct cancer cell apoptosis (Fig. 7F). These results suggest that EpAb2-6 has potential as a therapeutic antibody for tumor-targeted drug delivery, and imaging in cancer treatment.

## DISCUSSION

We have previously established that EpCAM (specifically the EpICD) promotes tumorigenesis in TICs through up-regulation of reprogramming genes and the epithelial-mesenchymal transition (EMT) process. The release of EpEX may further enhance EpCAM cleavage and trigger EpICD-mediated signaling in an autocrine or paracrine manner, which would consequently promote tumor initiation and progression [34]. Therefore, the development of therapeutic antibodies, which target EpCAM and/or inhibit EpICD activation, has great potential for eradicating tumors.

In this study, we developed a monoclonal antibody targeting EpCAM signaling and inhibiting EpICD activation in cancer cells. Our results indicate that EpAb2-6 can directly induce apoptosis in cancer cells by increasing cleavage of PARP and decreasing pro-caspase-3 proteins. To our knowledge, this is the first study to describe an anti-EpCAM mAb that directly induces cancer cell death by inhibiting EpCAM signaling, rather than by acting through the ADCC or CDC pathways. We also discovered that EpAb2-6 blocks EpICD cleavage and inhibits nuclear translocation of EpICD (Fig. 2E and 2F). Our results suggest that EpAb2-6 not only has potential as a therapeutic antibody for TICs, but it is also suitable for use in combinatorial treatment of CRC. Additionally, the present findings indicate that EpAb2-6 is a suitable diagnostic tool for the detection of CTCs and for early diagnosis of cancer via *in vivo* molecular imaging.

The mechanisms by which anti-EpCAM antibodies inhibit tumors *in vivo* remain unclear, due to inconsistent results arising from the use of EpCAM-specific antibodies as antineoplastic agents [61]. The antineoplastic effects of the anti-EpCAM antibody require the immune response; mAbs directed against EpCAM typically inhibit tumor growth via anti-idiotype networks, including both B and T cells, ADCC, and CDC death. Multiple trials assessing the efficacy of anti-idiotypic antibodies against EpCAM have achieved only marginal success [61]. No published study has demonstrated direct induction of cancer cell apoptosis by anti-EpCAM antibodies in a clinical setting, and so it remains unclear whether such antibodies can directly inhibit tumor cell proliferation [61]. In this study, EpAb2-6 was observed to directly induce apoptosis in HCT116 and HCT116 (*TP53^−/−^*) cell lines *in vitro* (Fig. 2) and inhibit CRC tumor growth (Fig. 5 and Supplementary Fig. S3) and metastasis (Fig. 6) *in vivo*. Our findings provide direct evidence that our anti-EpCAM antibody is able to inhibit tumor cell proliferation.

Our study found that p53 expression is increased by EpCAM knockdown [62]. Interestingly, EpAb2-6 also kills p53 mutant colon cancer cells (Table 2 and Supplementary Figs S2 and S3), suggesting that this antibody exerts its apoptotic effects through multiple mechanisms, potentially involving both p53-dependent and -independent pathways. Mutation or deletion of the *TP53* gene, which is associated with poor prognosis and drug resistance, is observed in over 50 percent of human tumors [63, 64]. This further highlights the importance of EpAb2-6 in cancer treatment. EpICD, the intracellular domain of EpCAM, has been reported to have a dominant role in EpCAM signaling [32]. Recent studies have reported accumulation of nuclear EpICD in tumor cells [65], tumorsphere-derived xenografts, and tumor tissues [34]. Blocking EpICD cleavage can prevent its nuclear translocation, and suppress reprogramming factors and expression of EMT genes [34]. In addition, Maaser *et al*. [66] suggested that EpCAM is involved in cell cycle-related signal transduction, which triggers intracellular signaling pathways. Sankpal *et al.* showed that p53 can bind to the *EpCAM* promoter to repress its activity [67]. Furthermore, recent studies have found that EpCAM up-regulation can enhance reprogramming of induced pluripotent stem cells by suppressing expression of p53 and p21, as well as maintaining the undifferentiated status of ESCs through control of pluripotent gene expression [35, 68]. In addition, it has been shown that c-Myc overexpression abrogates p21^CIP1^-mediated repression of EMT genes [69]. Other studies have shown that the activated Wnt/β-catenin pathway regulates EpCAM expression, indicating that EpCAM may be involved in the β-catenin-mediated self-renewal ability of tumor cells [70]. In line with these findings, we found that inhibiting EpCAM expression resulted in significantly higher levels of p53 and p21 proteins (data not shown). However, further investigation is required to elucidate the exact mechanism underlying inhibition of TIC tumorigenesis.

EpCAM is frequently overexpressed and functionally altered in malignant cells [6], including TICs and CTC [41]. Although normal epithelial tissues also express EpCAM, emerging evidence indicates that within normal epithelial tissues, membrane-bound EpCAM is largely sequestered within intercellular boundaries. Therefore, EpICD is not subject to cleavage in normal epithelial tissues, and it is also not observed in the nucleus [32, 34]. Dynamic changes observed in EpCAM expression have been linked to a changing tumor cell microenvironment during cancer progression [71]. EpCAM-positive CTCs are associated with poor prognosis, very low overall survival, and the presence of lymph node metastases [71, 72]. In order to verify whether EpAb2-6 has the ability to inhibit metastasis through mechanisms other than ADCC or CDC, we subjected cancer cells to non-attachment conditions to mimic CTCs. The results revealed that EpAb2-6 can increase the cleavage of capase-3 and PARP (Fig. 2D), thereby confirming EpAb2-6′s potential as a treatment for CTCs. Together, these findings suggest that EpCAM is required for the survival of CTCs in cases of colon cancer.

Despite effective adjuvant treatment, many patients experience disease recurrence and death from disseminated disease. Thus, there is a need for more effective adjuvant treatments for cancer. Colon cancer cells exhibit the highest frequency of high-level EpCAM expression of any cancer, with frequency >90% for any subgroup [6]. Such a high frequency of EpCAM expression on CRC at all stages of the disease makes colon cancer an ideal indication for anti-EpCAM-based therapies. The current standard treatment for stage IV metastatic CRC involves the use of a combination of chemotherapeutic agents, including irinotecan, fluorouracil, and leucovorin (IFL), following surgical resection [73]. In stage III colon cancer, adjuvant therapy with Edrecolomab plus Fluorouracil-based therapy had no statistically significant effect on the overall survival of patients [74].

Pancreatic cancer is one of the most deadly cancers, largely because it is often not diagnosed until the disease is at advances stages. Gemcitabine is the standard treatment for advanced and metastatic pancreatic cancer. The use of gemcitabine to treat pancreatic cancer tissue has had limited success, since it is poorly perfused and vascularized, and triggers a desmoplastic response [75]. In the past few years, several trials have been performed to investigate the efficacy of combination chemotherapy or targeted therapy for the treatment of pancreatic cancer, but no therapy resulted in strong enough results to replace gemcitabine monotherapy as the standard treatment [75, 76]. To evaluate the therapeutic efficacy of EpAb2-6 as a potential treatment for late stage colon and pancreatic cancers, we treated human colon cancer xenografts with either IFL alone or IFL in combination with EpAb2-6. Additionally, we also treated a pancreatic cancer metastatic animal model with either gemcitabine alone or gemcitabine in combination with EpAb2-6. Our results reveal that administration of EpAb2-6 not only enhances the antitumor activity of IFL against CRC, but it also increases the therapeutic efficacy of gemcitabine against pancreatic cancer. Importantly, EpAb2-6 markedly prolongs the median overall survival of metastatic tumor-bearing mice (Figs 5 and 6). Hence, EpAb2-6 may potentially increase the therapeutic index of the current metastatic CRC and pancreatic cancer treatment regimens when used in combination with IFL or gemcitabine.

While a plethora of antibodies target the well-described EGF-I of EpCAM, few are known to target the surface-exposed regions of the TY and EpICD [51]. To elucidate the exact mechanism underlying the inhibition of tumorigenesis by EpAb2-6, we used phage display to identify the B cell epitope of EpAb2-6: this revealed that positions Y95 and D96 in the EGF-II/thyroglobulin (TY) repeat (EGF-II/TY) domain of EpCAM are recognized by EpAb2-6 (Fig. 3). The ribbon and surface models show that the binding epitope of EpAb2-6 is different from that of the three anti-EpCAM antibodies currently in clinical trials (i.e., edrecolomab, ING-1, and adecatumumab). Interestingly, we found that the epitope of EpAb2-6 is localized in the TY loop and is very close to the cleavage site of β-secretase BACE1 (Beta-site APP Cleaving Enzyme) (22 Å). A recent study demonstrated that the release of EpICD from EpCAM triggers proliferation- and stemness-enhancing signaling in cancer cells [32, 34]. Moreover, the TY loop is known to help stabilize the cis-dimer architecture of two EpCAM molecules, which facilitate cell-to-cell contact [51]. We therefore hypothesize that the binding of EpAb2-6 may lead to steric hindrance, which disrupts cis-dimer formation on EpCAM and inhibits cleavage of EpEx by β-secretase. This in turn compromises the release of EpICD, ultimately causing cancer cell apoptosis.

In recent studies, EpEx was shown to form a cis-dimer corresponding to half of the proposed trans-tetrameric intercellular unit. The cis-dimer dimerization interface between the two subunits involves the TY domain [51]. TY domains are known for their potent inhibitory effects on cathepsins, and their involvement in metastasis and migration through the enhancement of extracellular matrix degradation [20, 77]. Several studies have investigated the role of EpCAM as a membrane-bound protease inhibitor, a function that may serve to protect tumor cells from their own secreted cathepsins during metastasis [20]. However, we have shown that treatment with sEpEX (soluble EpEX) or transfection with the EpEX gene promotes the cleavage of EpICD and induces activation of reprogramming genes, suggesting that EpEX cleavage may initialize EpCAM signaling, and its release may further activate EpCAM [34]. Maetzel et al. demonstrated that the shedding of ectodomain resulted in the formation of sEpEX, which is required as a ligand for EpCAM signaling [32]. Treatment of EpCAM-positive cells with a recombinant EpEX induced EpCAM cleavage, which indicated that soluble EpEX may provide cells with an autocrine or paracrine signal after its initial release trigger, similar to that of L1, epidermal growth factor (EGF) receptor, tumor necrosis factor (TNF) receptor, and others [78–81]. Therefore, the generation of inhibitors or antibodies against EpCAM may facilitate the development of tumor-targeting therapy (Figs 2, 5, and 6).

Based on our results, we propose that EpAb2-6 inhibits tumor growth through two pathways. The first is direct induction of cancer cell apoptosis or inhibition of EpCAM cleavage into EpICD and EpEX by binding to positions Y95 and D96 of the EGF-II/TY domain (Fig. 3) on intact EpCAM. The second is binding of EpAb2-6 to the neutralizing epitope on growth factor-like EpEX (Fig. 3), which subsequently prevents EpEX from binding to an unknown receptor. This in turn prevents the cleavage of EpCAM (Fig 2E and 2F). By inhibiting the cleavage of EpCAM, EpAb2-6 prohibits tumor growth by preventing the translocation of EpICD into the nucleus (Fig. 2F), thus preventing the binding of EpICD to a multiprotein complex consisting of FHL2, β-catenin, and Lef-1, and thereby blocking both the down-regulation of p53 and p21, and the activation of EMT and iPS genes.

The present study demonstrates that EpCAM may be a promising target antigen for the development of cancer therapy. EpAb2-6, which recognizes a particular epitope on EpCAM, can directly induce cancer cell death and may be a suitable basis for devising treatments for colon and pancreatic cancer.

## MATERIALS AND METHODS

### Cell lines and culture

The following human cell lines were used: oral cancer (SAS), nasopharyngeal carcinoma (NPC) [46], ovarian cancer cell line SKOV-3 (ATCC: HTB-77), colorectal cancer cell line HCT116 (ATCC: CCL-247), HCT116 (*TP53^−/−^*), or SW620 (ATCC: CCL-227) lung cancer cell line H441 (ATCC: HTB-174) or H1993 (ATCC: CRL-5909), breast cancer cell line MCF7 (ATCC: HTB-22), pancreatic cancer cell line BxPC-3 (ATCC: CRL-1687), PANC-1 (ATCC: CRL-1469) or AsPC-1 (ATCC: CRL-1682), 1112SK (ATCC: CRL-2429) and primary cultures of normal nasal mucosal epithelia (NNM). Primary cultures of NNM were generated from biopsies of patients with nasal polyposis [47]. The use of NNM was approved by the Human Subject Research Ethics Committee, Institutional Review Board, Academia Sinica (AS-IRB01-06008). Human umbilical vein endothelial cells (HUVECs) were purchased (Lonza, Walkersville, MD) and cultured in EBM-2 media (Lonza, Walkersville, MD). The human oral cancer cell line SAS was obtained from the Japanese Collection of Research Bioresources (Tokyo, Japan). The cells were cultivated in Dulbecco modified Eagle's media (DMEM) supplemented with 10% FBS, at 37°C with 5% CO_2_. Other cell lines were purchased from ATCC and were cultured in Dulbecco's Modified Eagle's media (DMEM) supplemented with 5% or 10% fetal bovine serum (FBS), at 37°C in a humidified incubator containing 5% CO_2_. Cells were cultured in accordance with protocols obtained from the ATCC and were passaged for fewer than 6 months after resuscitation.

### Generation of monoclonal antibodies and purification of IgG

Total HCT116 cell lysate protein was applied to an OCAb9-1 (an anti-EpCAM mAb)-coupled protein G sepharose 4 Fast Flow gel (GE Healthcare Biosciences, Pittsburgh, PA). The antibody-conjugated affinity columns were washed with PBS. The EpCAM proteins were eluted with elution buffer (Thermo Scientific, Rockford, IL) and neutralized with 1 M Tris-HCl, pH 9.1. The purified EpCAM protein was used to immunize mice for generation of mAbs against EpCAM. Anti-EpCAM mAbs were generated following a standard procedure [48], with slight modifications [49]. Briefly, female BALB/cJ mice (6-week-old, National Laboratory Animal Center (NLAC), Taipei, Taiwan) were immunized intraperitoneally with EpCAM protein four times at 3-week intervals. On day 4 after the final boost, splenocytes were harvested from the immunized mouse spleen and fused with NSI/1-Ag4-1 myeloma cells using 50% polyethylene glycol (GIBCO, CA, USA). Fused cells were suspended in DMEM supplemented with hypoxanthine-aminopterin-thymidine (HAT) (Sigma, St. Louis, MO) and hybridoma cloning factor (ICN, Aurora, Ohio), and were then plated onto 96-well plates. These hybridomas, which were positive for HCT116 and SAS but negative for NNM, were then subcloned by limited dilution, before being preserved in liquid nitrogen. Ascites were produced in pristane-primed BALB/cJ mice, and mAbs were purified using a protein G Sepharose 4G gel (GE Healthcare Biosciences, Pittsburgh, PA). The protocol was approved by the Committee on the Ethics of Animal Experiments of Academia Sinica (AS IACUC: 11-04-166).

### Western blot analysis

For Western blotting, cells were extracted using RIPA buffer (0.01 M sodium phosphate (pH 7.2), 150 mM NaCl, 2 mM EDTA, 50 mM NaF, 1% Nonidet P-40, 1% sodium deoxycholate, and 0.1% SDS) containing protease inhibitor cocktail (Roche, Indianapolis, IN). Equal amounts of protein were separated by SDS-PAGE and then transferred to PVDF membranes (Millipore, Bedford, MA, USA). The membrane was blocked with 1% BSA, and incubated with anti-EpCAM (1 μg/ml), biotinylated polyclonal goat anti-human EPCAM (R&D Systems BAF960), biotin-conjugated anti-human CD326 (clone 9C4, Biolegend #324316), anti-human epithelial cell antigen-specific antibody (clone VU-1D9; Sigma), anti-EpICD mAb (1:100 dilution; 1144-1; Epitomics, Burlingame, CA), PARP (sc-8007, Santa Cruz Biotechnology, INC), or Caspase3 (Asp175, 5A1E, Cell Signaling) mAbs overnight. Membranes were then incubated with HRP-conjugated secondary antibodies (1:5000, Jackson Immunoresearch) for 1 hour at room temperature, and protein expression was detected using an ECL kit (Millipore, Temecula, CA, USA).

### Immunofluorescent staining

Cells cultured on cover slips were fixed in 2% paraformaldehyde for 20 min before being washed, and subsequently blocked with 1% bovine serum albumin in PBS for 30 min. Cells were incubated at room temperature with anti-EpCAM mAbs (1 μg/ml) or anti-EpICD mAb (1:100 dilution; 1144-1; Epitomics, Burlingame, CA), in 1% bovine serum albumin. After 1 hour incubation, cells were washed and incubated with Alexa Fluor 488 goat-anti-mouse (Invitrogen), or Alexa Fluor 568 goat-anti-rabbit (Invitrogen) antibodies. Nuclei were counterstained with 4′, 6-Diamidino-2-phenylindole (DAPI 1:500).

### Flow cytometry

SAS, HCT116, and NNM cells were dissociated with 0.25% trypsin-EDTA (1 mM) (Invitrogen) for 1–3 min. Cells were washed with cell sorting buffer (PBS containing 1% fetal calf serum), and then incubated for 1 hour at 4°C in cell sorting buffer with anti-EpCAM mAbs at dilutions ranging from 0.00001 to 1 μg/ml. Cells were then incubated with phycoerythrin-conjugated goat anti-mouse IgG (dilution 1:250; Jackson ImmunoResearch Laboratories, West Grove, PA) for 30 min at 4°C. After a final wash, the cells were re-suspended with 1% FBS in PBS and analyzed by flow cytometry (BD, San Jose, CA).

### Apoptosis assays

Cells were separately seeded and treated with 0–20 μg/ml mAbs for 6 hours; an unrelated mouse myeloma immunoglobulin served as the IgG2a (Invitrogen #02-6200) isotype control at an appropriate dilution. Apoptotic cells were detected using Annexin V-FITC and PI, and were analyzed using a flow cytometer (BD immmunocytometry systems, San Jose, CA). Early apoptosis was measured with the Annexin V-FITC Apoptosis Detection kit II (BD Pharmingen, La Jolla, CA). Apoptotic nuclei were detected with propidium iodide (PI) staining.

### MTT assay

The effect of EpAb2-6 on cell viability was assessed using MTT assays (Thiazolyl Blue Tetrazolium Bromide; Sigma, USA) according to the manufacturer's protocol. To perform the MTT assay, cells were mixed in the presence of increasing concentrations of EpAb2-6 (1, 2, 4, 8, 12, 20 μg/ml), in a final volume of 150 μl 2% FCS culture media. The same concentration of IgG was used as a control. The serially diluted EpAb2-6 antibodies or control IgG were then plated onto flat-bottomed 96-well plates at a density of 5 × 10^2^ cells/well. After 48 h of incubation, 1 μl of MTT reagent was pipetted into each well of the 96-well assay plate to a final concentration of 5 μg/ml (wells contained 100 μl of fresh phenol red-free culture media). The plate was then incubated for 2 h at 37°C in a humidified, 5% CO2 atmosphere, and media were subsequently replaced with 150 μl of DMSO. The absorbance (A) at 570 nm was then recorded using a Spectra^®^ Max M5 Series (Molecular Devices). Cellular viability was calculated as (Asample − Ablank) / (Acontrol − Ablank) × 100%. All experiments were repeated at least three times, with triplicate samples for each experiment.

### Non-attachment cell death assay

Cells were plated at 5 × 10^2^ per 25T flask in 4 ml growth media at the indicated time points. The cells were subsequently incubated on a shaker at 130 rpm at 37°C in a humidified, 5% CO_2_ atmosphere. Cells in suspension were collected and washed with 1 × PBS, and were then lysed in RIPA buffer for subsequent protein analysis by Western blotting.

### Identification of B-cell epitopes of EpAb2-6 by phage display

The phage display biopanning procedures were performed as described previously [50]. Briefly, an ELISA plate was coated with mAb at 100 μg/ml by incubation at 4°C for 6 h. After washing and blocking, the phage-displayed peptide library (New England BioLabs, Inc.) was diluted to a concentration of 4 × 10^10^ pfu, and incubated for 50 mins at room temperature. After washing, bound phage was eluted with 100 ml of 0.2 M glycine/HCl (pH 2.2) and neutralized with 15 ml of 1 M Tris/HCl (pH 9.1). The eluted phage was amplified in ER2738 (New England Biolabs, Inc. MA, USA) for subsequent rounds of selection; the phage was then titrated on LB/IPTG/X-Gal plates. The biopanning protocols for the second and the third rounds were identical to the first round, except for the addition of 2 × 10^11^ pfu of amplified phage. The immunopositive phage clones were identified by ELISA, and then sequenced with the −96 primer 5′-CCCTCATAGTTAGCGTAACG-3′ (this primer corresponds to the pIII gene sequence of M13 phage). The phage-display peptide sequences were translated using the ExPASy Proteomics Server and aligned using MacDNAsis software.

### Use of EpCAM mutants to identify the B-cell epitope of EpAb2-6

Site-directed mutagenesis was used to generate EpCAM mutants, using the recombinant expression plasmid pcDNA™ 3.1/V5-His. PCR was performed using pfu ultra DNA polymerase (MERCK), and all mutant constructs were confirmed by sequencing. HEK293 cells at 80–90% confluency in 6-well plates were transfected with plasmids encoding various EpCAM mutants. After two days of transfection, the cells were washed with PBS. Cells were extracted with RIPA buffer, supplemented with a protease inhibitor mixture tablet, and centrifuged at 20,000 g for 30 min at 4°C. The wild-type and mutated recombinant proteins were stained by incubating with 1 μg/ml primary antibody (EpAb2-6 or EpAb3-5), followed by HRP-conjugated secondary antibodies (Jackson Immuno Research Labs, West Grove, PA). The signals were developed using enhanced chemiluminescence reagents (ECL) (Thermo Scientific, Rockford, IL).

### Construction of an EpCAM deletion mutant

PCR-amplified human EpCAM was cloned into pcDNA3.1-v5-His, and the EGF-like domain I (amino acids 27 to 59) and II (amino acids 66 to 135) of full-length EpCAM were removed using PCR-based gene deletion. Each construct was confirmed by DNA sequencing. Primer sequences used for PCR mutagenesis are listed in Supplementary Table S1.

### Synthesis of EpAb2-6-HiLyte-750 conjugated (EpAb2-6-HL750) and imaging

EpAb2-6 or NM-IgG (600 μg) were incubated with HEPES solution containing 20 nmole HiLyte Fluor™ 750 acid NHS ester (HiLyte-750) (AnaSpec) at 4°C overnight, in order to conjugate HiLyte-750 to the mAb via the NHS functional group. EpAb2-6- HiLyte-750 conjugated was purified using a NAP-10 column with HEPES buffer. The concentration of HL750 was determined using a spectrofluorometer and interpolation from a standard curve. The mice were randomly divided into three groups (3 mice in each group), for intravascular injection with control (HiLyte Fluor™ 750 dye only), NM-IgG-HL750, or EpAb2-6-HL750. Fluorescence imaging was performed using Xenogen's IVIS^®^ 200 imaging system (Excitation: 710/760 nm; Emission: 810/875 nm) at the indicated times.

### Animal model for analysis of antitumor efficacy

NOD/SCID mice were obtained from the Jackson Laboratory (Bar Harbor, Maine, USA) and were bred in the core facility of the ICOB at Academia Sinica. Mice of 4–6 weeks old were injected subcutaneously in the dorsolateral flank with 2 × 10^6^ HCT116 cells. Mice with size-matched tumors (approximately 50 mm^3^) were then randomly assigned to different treatment groups, and were injected with EpAb2-6 only, IFL only, EpAb2-6 plus IFL, or equivalent volumes of saline through the tail vein. For EpAb2-6 monotherapy, EpAb2-6 was delivered at a dose of 20 mg/kg through the tail-vein by intravenous (i.v.) injection twice a week for 4 weeks. For IFL monotherapy, IFL (fluorouracil at 25 mg/kg + leucovorin at 10 mg/kg + irinotecan at 10 mg/kg) were administered by i.v. injection twice a week for 4 weeks. For combination treatment, EpAb2-6 was administered 24 hours before IFL; EpAb2-6 and IFL were administered at the same dosage as the monotherapy groups. Mouse body weight and tumor size were measured twice a week. Tumor volumes were calculated using the equation: length × (width)^2^ × 0.52 and presented as standard error of the mean. This study used humane endpoint by determining the tumor size (>10% of body weight) or mouse weight loss (>20% of body weight). Mice were observed twice a week and were given a soft diet. Animal care was carried out in accordance with the guidelines of Academia Sinica, Taiwan. The protocol was approved by the Committee on the Ethics of Animal Experiments of Academia Sinica (AS IACUC: 11-04-166).

### Colon and pancreatic cancer metastatic animal model

HCT116 colon cancer cells (1 × 10^6^ cells/mouse) were injected into 6-week-old female NOD/SCID mice (Jackson Laboratory) through the lateral tail vein. Mice were then treated with PBS and EpAb2-6. The dosage was 20 mg/kg on days 1 and 4. Mouse body weight and survival rate were measured twice a week (*n* = 10). For EpAb2-6 monotherapy, EpAb2-6 was delivered at a dose of 20 mg/kg through the tail-vein by intravenous (i.v.) injection once a week for 5 weeks. IFL was administered by intravenous (i.v.) injection once a week for 5 weeks. AsPC-1 pancreatic cancer cells (1 × 10^6^ cells/mouse) were injected into NOD/SCID mice through the lateral tail vein. Mice were then treated with PBS, isotype (IgG2a), or EpAb2-6. The dosage was 20 mg/kg on days 1, 8, 15, 22, and 29. Mouse body weight and survival rate were measured twice a week (*n* = 10). For EpAb2-6 monotherapy, EpAb2-6 was delivered at a dose of 20 mg/kg through the tail-vein by intravenous (i.v.) injection once a week for 5 weeks. Gemcitabine monotherapy at 80 mg/kg was administered by intraperitonel (i.p.) injection once a week for 5 weeks. Mice were monitored every day and given a soft diet to decrease the suffering of the mice. For the study of mouse survival, tumor size was not appropriate for use as a humane endpoint. Instead, humane endpoints were decided based on mouse weight loss (>20% of body weight) or mouse activity assessment (hunching, stationary, ruffling, and poor grooming). Animal care was carried out according to the guidelines established by Academia Sinica, Taiwan. The protocol was approved by the Committee on the Ethics of Animal Experiments of Academia Sinica (AS IACUC: 11-04-166).

### Cloning and CDR sequencing of anti-EpCAM antibodies

Total RNA was extracted from hybridoma cells using TRIzol reagent (Invitrogen), and mRNA was isolated with the NucleoTrap mRNA Mini Kit (Macherey-Nagel GmbH & Co. KG.). Purified mRNA was reverse transcribed using oligo (dT) as a primer in a ThermoScript RT-PCR system (Invitrogen). The variable heavy- and light-chain domains (V_H_ and V_L_) were amplified from the cDNA product by PCR with a variety of primer sets (Dubel et al., 1994; Orlandi et al., 1989; Orum et al., 1993). The PCR products were cloned using the TA kit (Promega, Madison, WI), and the V_H_ and V_L_ sequences were determined by DNA sequencing. Software Vector NTI (InforMax) was used for sequence analysis. From these sequences, the framework regions (FR) and complementarity-determining regions (CDR) were analyzed through comparison with those found in the Kabat database and with alignment to sequences in the ImMunoGeneTics database (Lefranc et al., 2009).

### Construction and expression of humanized EpAb2-6

Humanized EpAb2-6 V_H_ consisted of the modified FR1 to FR4 from the accession DI164282 gene, and the CDR1 to CDR3 of EpAb2-6 V_H_, respectively. The humanized EpAb2-6 V_L_ CDRs consisted of the modified FRs from the accession GM882764 gene and the CDRs of EpAb2-6 V_L_. The resulting V_H_ was cloned into modified expression vector pcDNA3.1 (Invitrogen) with a signal peptide and human IgG1 constant region. The V_L_ was cloned into modified expression vector pSecTag (Invitrogen). The V_H_ and V_L_ plasmids were cotransfected into CHO-K1 cells and selected using G418 and puromycin for 2–3 weeks. Transformed cells were subjected to limiting dilution in 96-well plates. After two weeks, stable clones produced humanized antibody in McCoy's 5A medium (Sigma-Aldrich), and were identified by ELISA. Humanized antibodies were produced by CELLine AD 1000 (INTEGRA Biosciences, Switzerland), according to the manufacturer's recommendations.

### Statistical analysis

All data were derived from at least three independent experiments. Values are expressed as the mean ± SD. Experimental test conditions were compared with the respective control by Student's *t*-test, unless otherwise specified. Differences were considered significant at *p value < 0.05, **p value < 0.01, or ***p value < 0.001. Survival analyses were performed using Kaplan-Meier survival curves, and significant differences between groups were tested using the log-rank test. Correlation coefficients were assayed by Spearman's analysis.

## SUPPLEMENTARY SUPPORTING INFORMATION FIGURES AND TABLE


