# Supplementary material for: An anti-EpCAM antibody EpAb2-6 for the treatment of colon cancer
Source: Oncotarget. 2015 Aug 6;6(28):24947–68. doi: 10.18632/oncotarget.4453 (PMC4694806; doi:10.18632/oncotarget.4453)
Supplement: Supplementary file 1 [file oncotarget-06-24947-s001.pdf]

SUPPORTING INFORMATION

LENTIVIRUS PRODUCTION

Small hairpin RNA vectors for EpCAM silencing (5′-GCAAATGGACACAAATTACAA-3′; 5′-GCCGTAAACTGCTTTGTGAAT-3′) were obtained from the National RNAi Core Facility (Academia Sinica, Taiwan). To generate stable EpCAM-knockdown cell lines, HEK293T packaging cells were co-transfected with a packaging plasmid (pCMV-ΔR8.91), envelope (pMDG) and hairpin pLKO-RNAi vectors using a PolyJET Transfection Kit (SignaGen Laboratories, Ijamsville, MD, USA). At 48 h post-transfection, virus-containing supernatants were collected, mixed with fresh media containing polybrene (8 μg/ml), and incubated with target cells for another 48 h. Transduced cells were selected with puromycin (2 μg/ml) for 7 days.

ELISA

Cell culture (96-well) plates (Corning Costar, St Louis, MO) were seeded with SAS, NPC, H441, HCT116, SKOV-3, MCF7, BxPC-3, NNM, or HUVEC cells. The plates were fixed with 2% paraformaldehyde, and blocked with 1% bovine serum albumin. OCAb9-1 was added to the plates, which were then incubated for 1 hour. The plates were subsequently washed with PBS containing 0.1% (w/v) Tween 20 ($PBST_{0.1}$), followed by incubation with horseradish peroxidase-conjugated anti-mouse IgG (Jackson ImmunoResearch Laboratories) for 1 hour. After washing, the plates were incubated with substrate solution (*o*-phenylenediamine dihydrochloride, Sigma). The reaction was stopped by the addition of 3 N HCl, and signals were detected using a microplate reader at 490 nm.

SURFACE PLASMON RESONANCE

The affinities of murine and humanized antibodies were determined by surface plasmon resonance (BIAcore T200, Biacore, Inc). EpCAM antigen was immobilized on a Series S Sensor Chip CM5 (Biacore, Inc) and injected at a flow rate of 10 μl/min. The mAbs were diluted in HBS-EP+ buffer (Biacore, Inc) and injected at a flow rate of 30 μl/min for 1.5 min; mAbs were then allowed to dissociate over 5 min. Before injection of each mAb, the surface was regenerated by injection of a solution of 10 mM glycine HCl, 0.2 M NaCl (pH2.5). The data were analyzed using BIAevaluation software with a global fit 1:1 binding model.

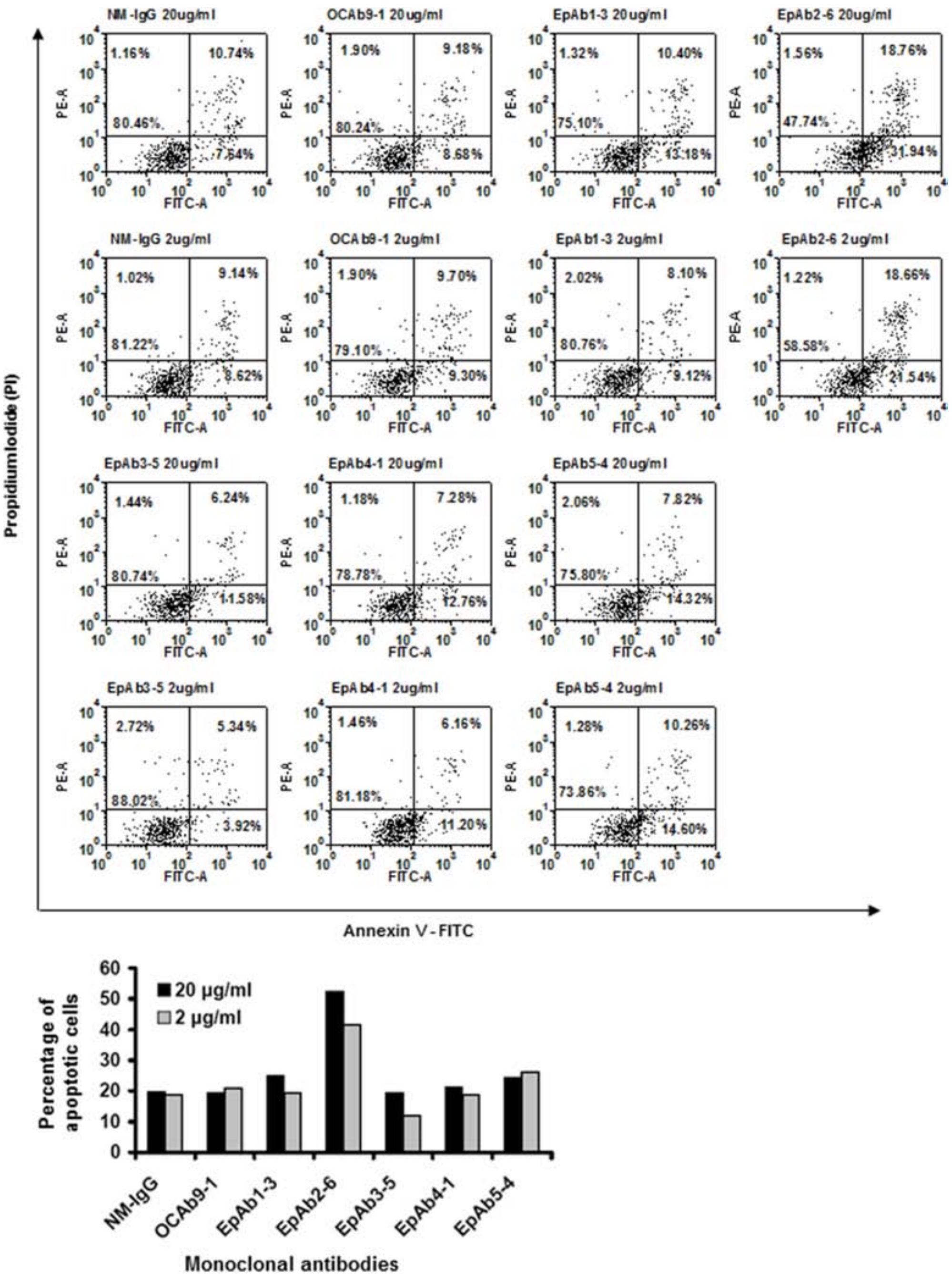

Supplementary Figure S1: Inhibition of cancer cell growth *in vitro* by EpAb2-6. SAS cells were treated with NM-IgG, OCAb9-1, EpAb1-3, EpAb2-6, EpAb3-5, EpAb4-1, or EpAb5-4 (2 and 20 μg/ml) for 6 hours, and cell death was measured by flow cytometry with Annexin-V FITC and PI double staining.

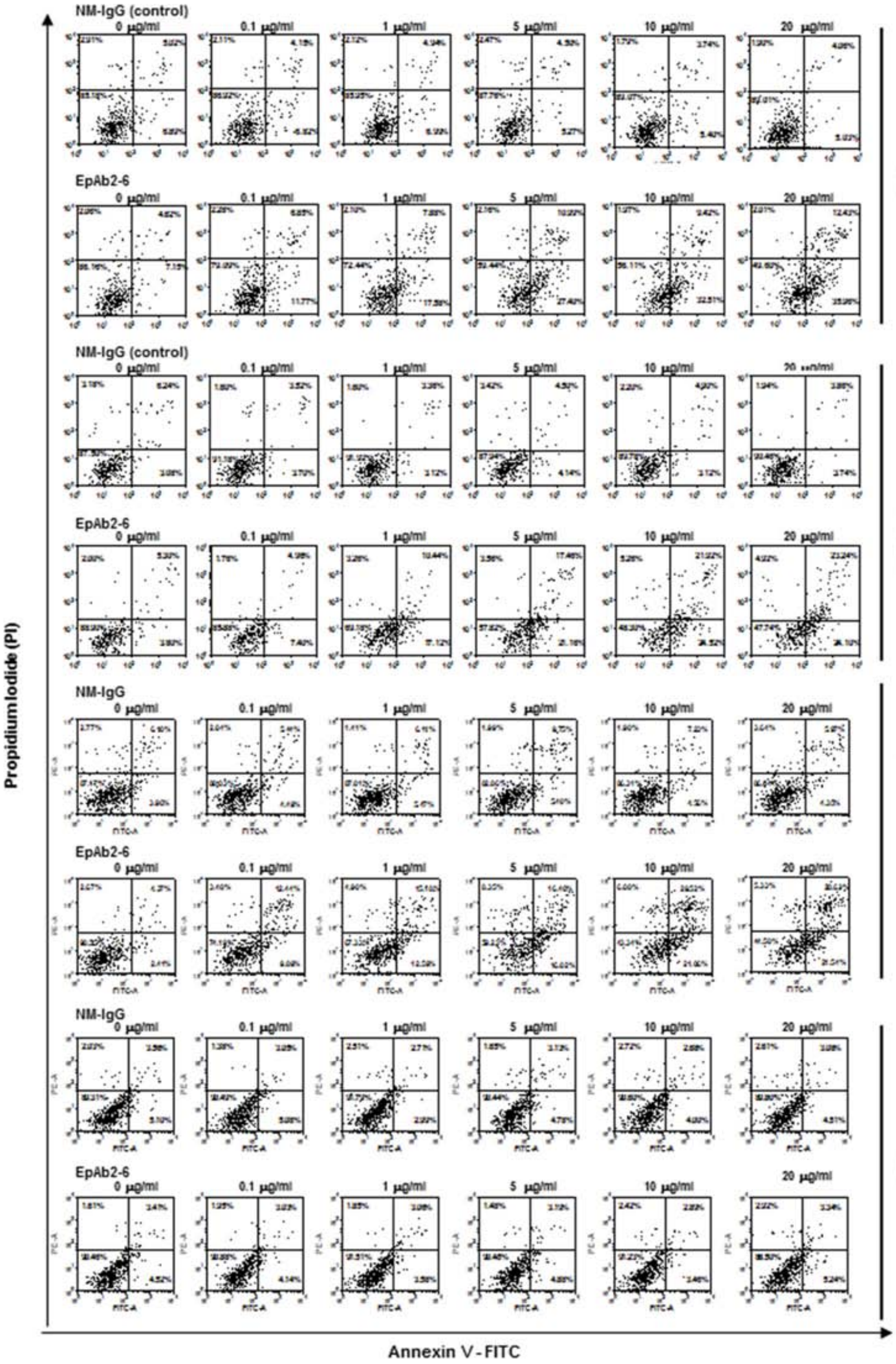

Supplementary Figure S2: Induction of apoptosis *in vitro* by EpAb2-6. SAS, HCT116, HCT116 (*TP53*$^{-/-}$), and NNM cells were treated with EpAb2-6 (0–20 μg/ml) for 6 hours, and cell death was measured by flow cytometry with Annexin-V FITC and PI double staining.

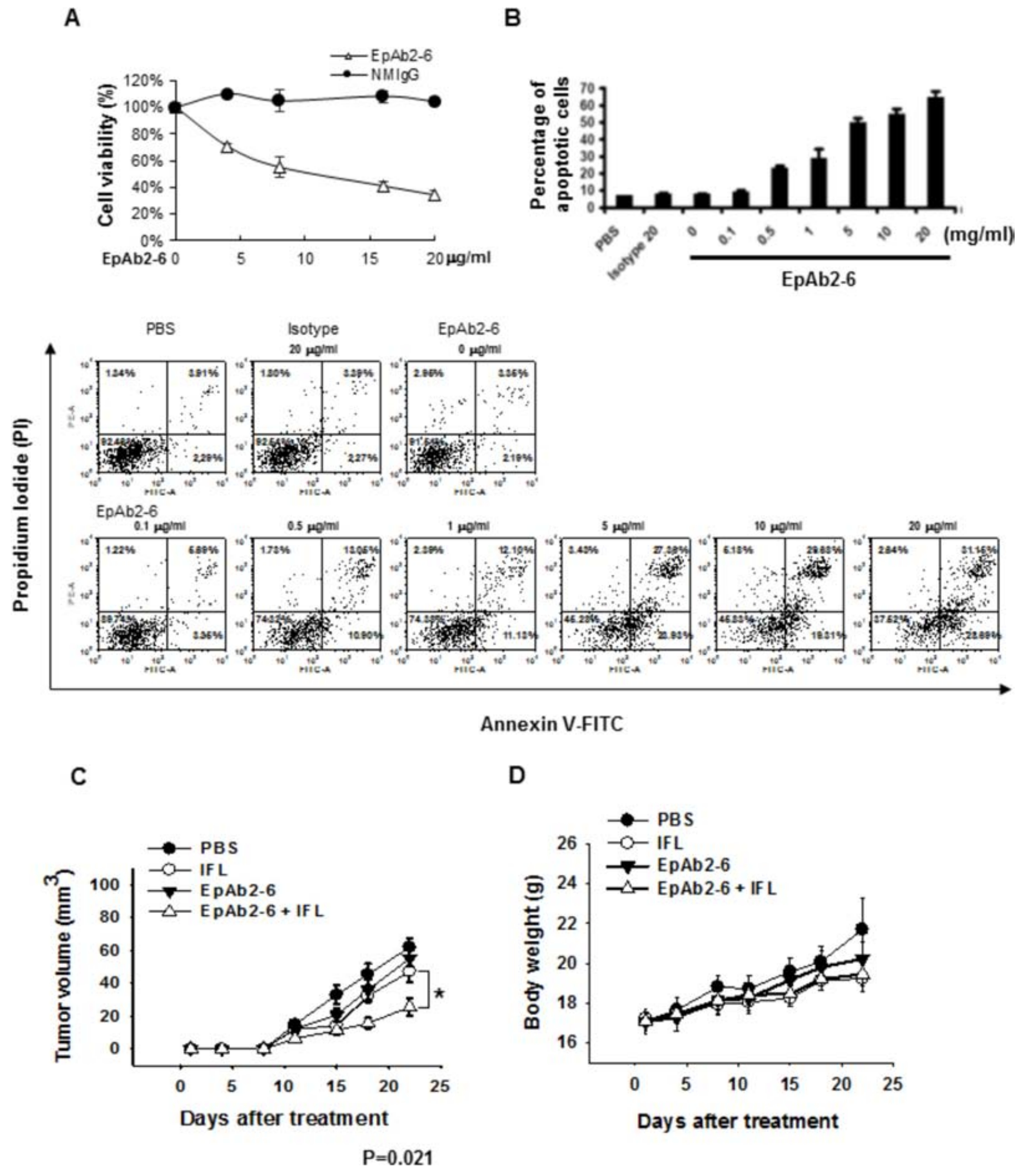

Supplementary Figure S3: Inhibition of colon cancer cell SW620 growth *in vitro* and *in vivo* by EpAb2-6. A. Cell viability assay. SW620 cells were treated with EpAb2-6 (0–20 µg/ml) for 48 h. Error bars show mean ± SD. **B.** SW620 cells were treated with EpAb2-6 (0–20 µg/ml) for 6 hours, and cell death was measured by flow cytometry with Annexin-V FITC and PI double staining. **C.** Mice bearing SW620-derived tumor xenografts were treated with EpAb2-6, IFL, EpAb2-6 in combination with IFL, or PBS. The sizes of tumors in each group were determined on the indicated days. Error bars show mean ± SD ($n = 6$) (Student's t-test, $*p < 0.05$). **D.** Average body weight of each group is shown on the indicated days. Error bars show mean ± SD.

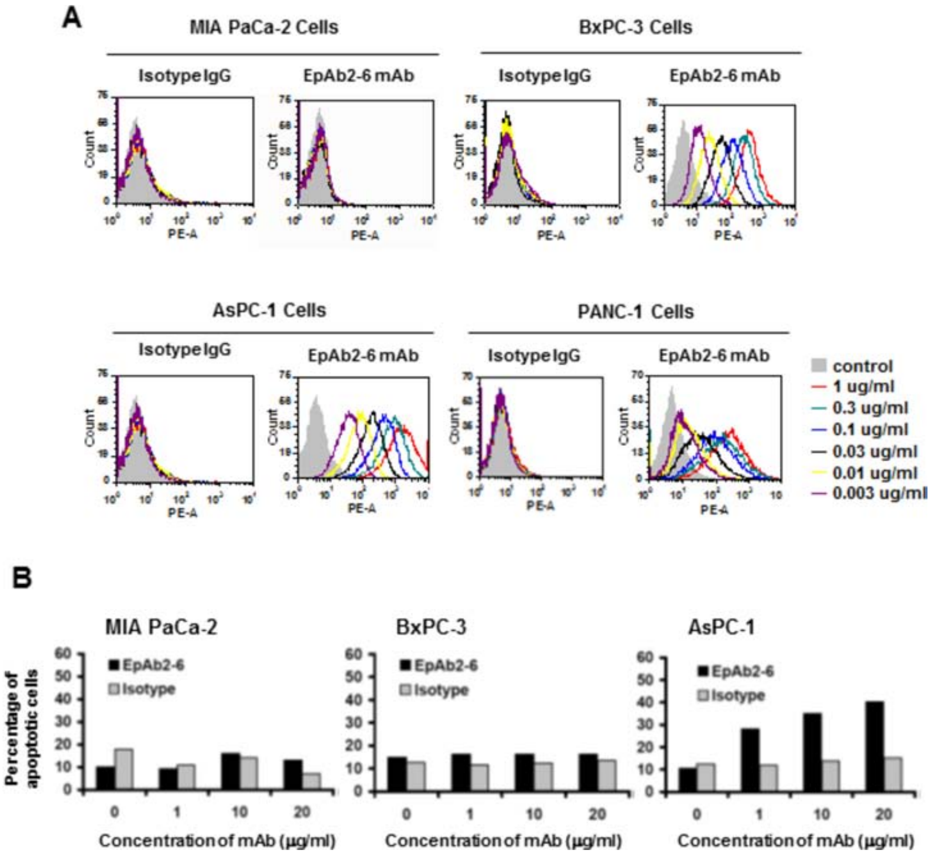

Supplementary Figure S4: Inhibition of pancreatic cancer cell growth *in vitro* by EpAb2-6. Flow cytometry was used to show that anti-EpCAM antibody can bind to AsPC-1, BxPC-3, and PANC-1 cells, but not to MIA PaCa-2 cells **A.** MIA PaCa-2, BxPC-3, and AsPC-1 cells were treated with EpAb2-6 (0–20 μg/ml) for 6 hours, and cell death was measured by flow cytometry with Annexin-V FITC and PI double staining.

Supplementary Table S1. Primers used for cloning and PCR-based site directed mutagenesis of EpCAM

Assay	Gene	Sequence (5′→3′)
Cloning primers	*EpCAM*	F: GATAAGCTTATGGCGCCCCCGCAGGTC<br>R: GATCTCGAGTGCATTGAGTTCCCTATGCATCTCACC
Deletion mutation primers	*EGF I*	F: AAAGGTACCTCAAAGCTGGCTGCCAAATG<br>R: AAAGGTACCTTCTTCCTGAGCTGCGGC
	EGF II	F: AAAGGTACCTCTGAGCGAGTGAGAACC<br>R: AAAGGTACCTTTGGCAGCCAGCTTTGAG
